# Cerium Containing Bioactive Glasses: A Review

**DOI:** 10.1021/acsbiomaterials.1c00414

**Published:** 2021-09-01

**Authors:** Alfonso Zambon, Gianluca Malavasi, Annalisa Pallini, Francesca Fraulini, Gigliola Lusvardi

**Affiliations:** Department of Chemical and Geological Sciences, University of Modena and Reggio Emilia, via Campi 103, 41125 Modena, Italy

**Keywords:** BGs, cerium, bioactivity, cellular activity, ROS

## Abstract

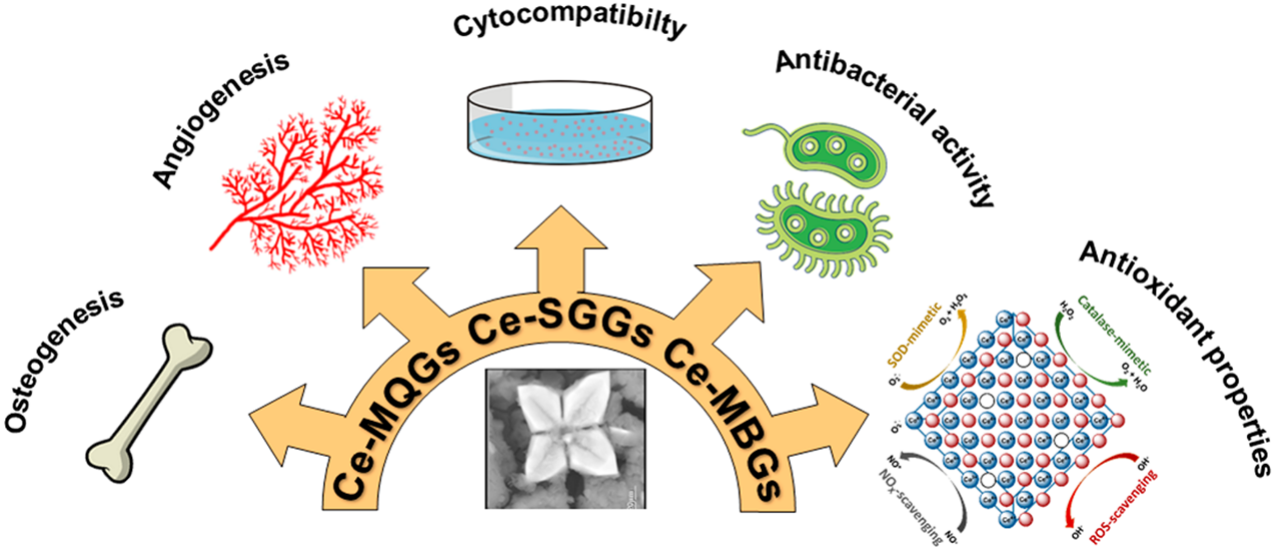

Bioactive glasses (BGs) for biomedical applications are doped with therapeutic inorganic ions (TIIs) in order to improve their performance and reduce the side effects related to the surgical implant. Recent literature in the field shows a rekindled interest toward rare earth elements, in particular cerium, and their catalytic properties. Cerium-doped bioactive glasses (Ce-BGs) differ in compositions, synthetic methods, features, and *in vitro* assessment. This review provides an overview on the recent development of Ce-BGs for biomedical applications and on the evaluation of their bioactivity, cytocompatibility, antibacterial, antioxidant, and osteogenic and angiogenic properties as a function of their composition and physicochemical parameters.

## 1 Introduction

The treatment of bone injuries from trauma or disease requires materials with specific mechanical and chemical properties.^1^ Among them, bioactive glasses (BGs) have been widely used for the treatment of bone defects due to their ability to bond and integrate with the soft and hard tissues of the living body.

This property is associated with the formation of a hydroxycarbonate apatite (HCA) layer on the surface of the glass, following initial glass dissolution. HCA is similar to mineral bone and is thought to interact with collagen fibrils to bond with the host bone; in this process, the release of active ions from the BGs is paramount for bone regeneration.^2^

**Figure 1:**
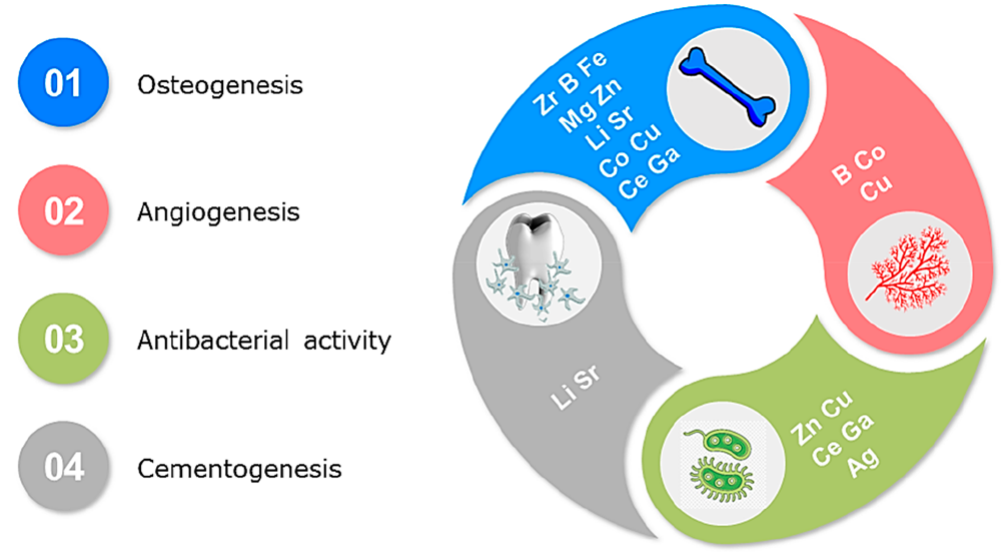
Biological effects of the addition of TIIs to BGs.

The first BG (45S5 Bioglass, hereafter abbreviated as 45S5) was developed in 1969^1,3,4^ with a weight composition of 45% SiO_2_, 24.5% Na_2_O, 24.5% CaO, and 6% P_2_O_5_. “Bioglass” was trade-marked by the University of Florida for the 45S5 composition.^4^ BGs were doped with therapeutic inorganic ions (TIIs)^5−8^ (Figure 1) to improve their properties and reduce postimplantation problems and thus the need for lengthy drug treatments and long recovery times. The addition of TIIs to the BG composition can improve the osteogenesis, angiogenesis, antibacterial activity, and cementogenesis of the material.^5^

**Figure 2:**
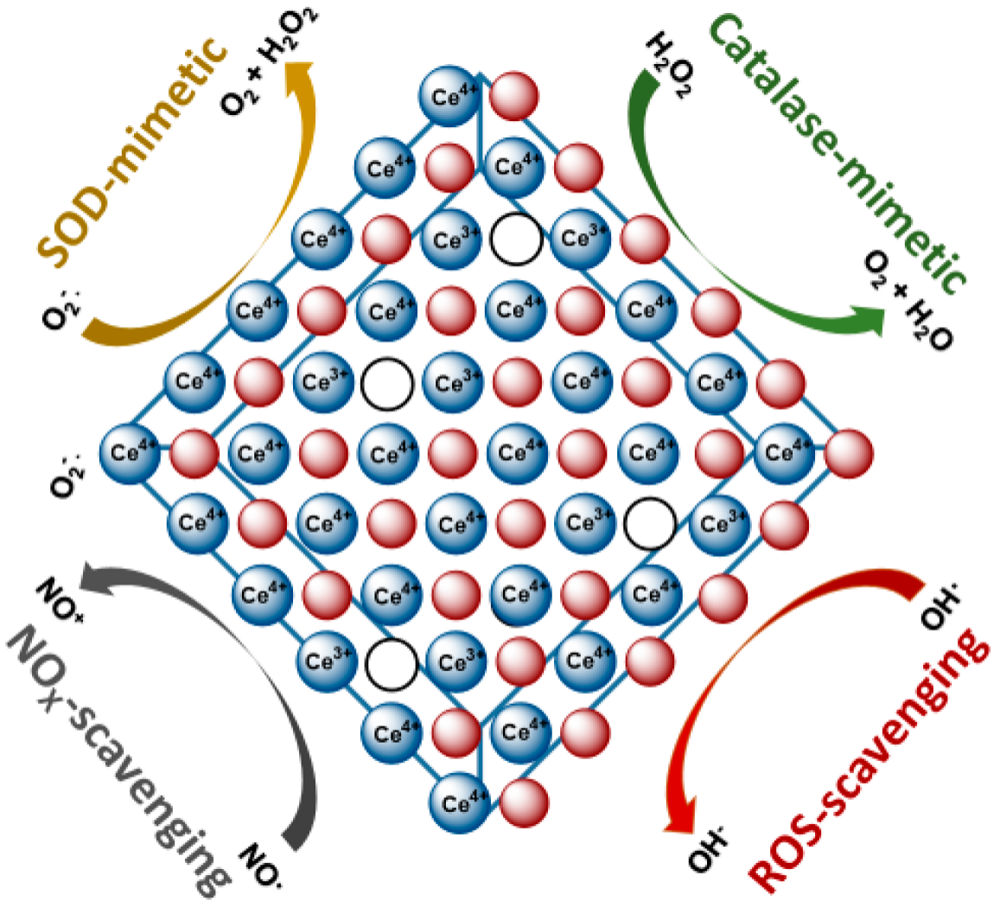
Antioxidant properties of CeNPs.

The rekindled interest toward rare earth elements and toward cerium and its catalytic properties in particular prompted the investigation of cerium and its compounds for therapeutic applications.^9−11^ Cerium compounds have been known for some time to have relevant pharmacological properties^12^ and have been used, for example, as antiemetics,^13^ bacteriostatics,^14^ and antitumorals.^15^ There are nevertheless limitations to the use of such compounds in biomedicine, namely, their nonspecific biodistribution, limited cell permeability, and low solubility.^16^ These limitations can be overcome by the use of cerium oxide nanoparticles (CeNPs),^17^ which are cell permeable and can be potentially targeted to specific tissues; furthermore, their solubility can be modulated by coating the material with water-soluble polymers.^15,18^ CeNPs can act as both oxidation and reduction catalyst, depending on the Ce^3+^/Ce^4+^ ratios and the oxygen defects on the surface.^19^ Their redox activity is due to the quick alternation between the two oxidation states. CeNPs can thus act as a multienzyme mimic or radical scavengers (Figure 2) by dismutating or scavenging reactive oxygen species (ROS) and reactive nitrogen species (RNS).^19^ In the former case, the Ce^3+^/Ce^4+^ surface ratio is critical in determining the profile of the system, as CeNPs with high Ce^3+^/Ce^4+^ surface ratios are effective at catalyzing the dismutation of the superoxide anion O_2_^•–^ (superoxide dismutase (SOD) mimetic activity), while CeNPs with low Ce^3+^/Ce^4+^ surface ratios are effective at catalyzing the dismutation of H_2_O_2_ (catalase (CAT) mimetic activity). Furthermore, CeNPs can act as scavengers of other ROS such as the peroxide radical (OH^•^) and RNS like the nitric oxide radical ^•^NO.^20^

The toxicity of NPs in general is a major concern for their biomedical applications.^21^ Although still controversial, CeNPs are generally considered as low toxicity or biocompatible materials.^22^ CeNPs are thought not to induce DNA damage or genotoxicity at certain doses.^16^ However, there are also a few reports suggesting that these NPs may be toxic depending on their shape, size, and oxidative status. Their *in vivo* ADMET (adsorption, distribution, metabolism, excretion, and toxicity) behavior needs thus to be carefully investigated before their biomedical application is granted.^16,23,24^

Conversely, conventional BGs doped with TIIs represent a viable therapeutic strategy for the treatment of a range of conditions and diseases and are routinely used in otology, orthopedics, and dentistry.^5,25,26^ Other potential applications include treatment of ear diseases (1977, *in vivo* and clinical trial),^27^ treatment of liver cancer (1987, *in vivo* and clinical trial),^28^ peripheral nerve repair (1998, *in vivo*),^29^ wound healing (2000, *in vivo*),^30^ lung tissue engineering (2004, *in vitro*),^31^ skeletal muscle and ligament repair (2005, *in vitro*),^32,33^ gastrointestinal applications (2005, *in vitro*),^31^ cardiovascular tissue engineering (2010, *in vitro*),^34^ embolization of uterine fibroids (2012, *in vitro* and *in vivo*),^35^ spinal cord repair (2012, *in vivo*),^36^ and treatment of metastatic colorectal carcinoma of the liver (2018, clinical trial).^37^

The recent literature reports several detailed studies on cerium-doped bioactive glasses (Ce-BGs) that differ by compositions, synthetic methods, features, and *in vitro* tests. The purpose of this review is to provide a critical overview on the development and applications of Ce-BGs.

## 2 Synthesis

**Figure 3:**
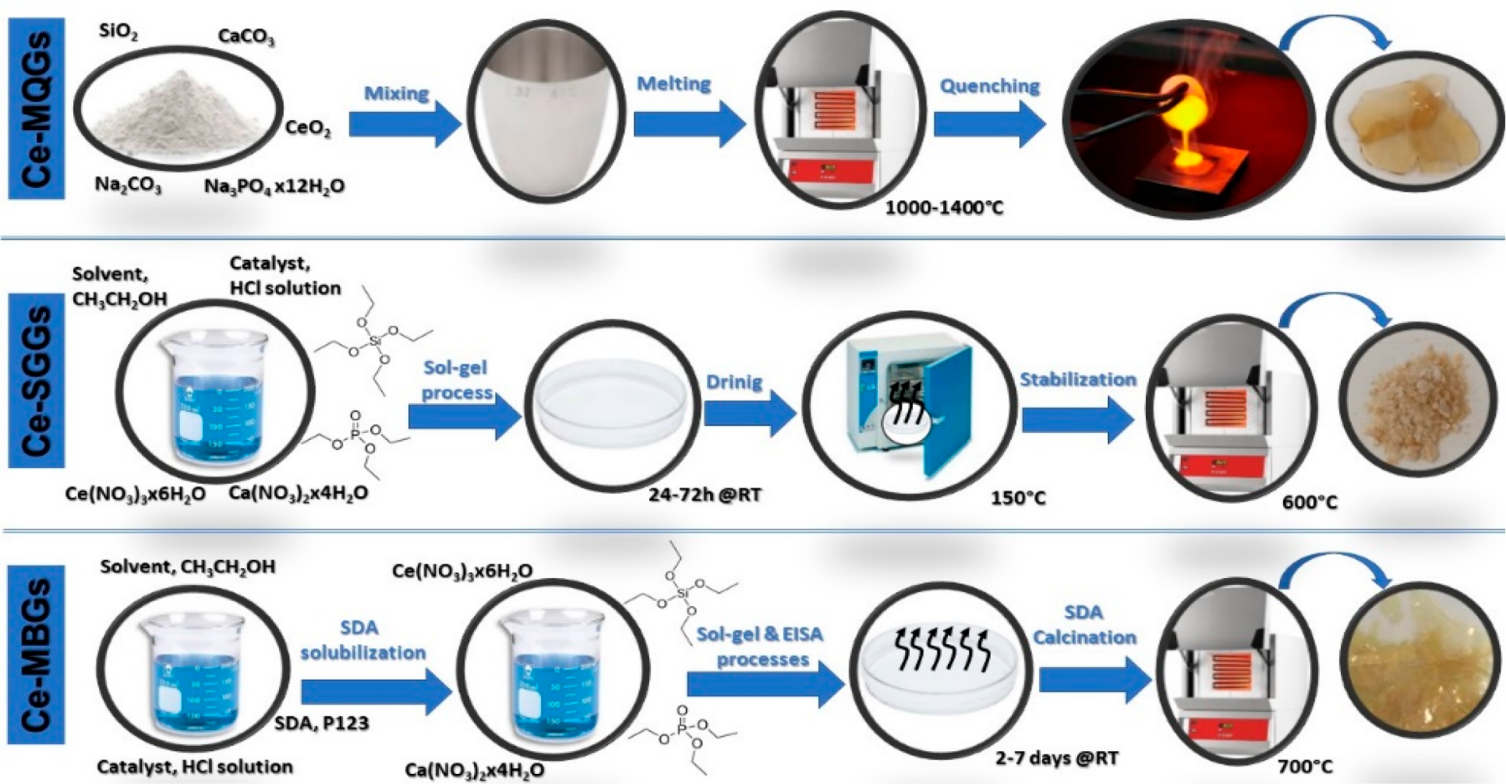
Schematic representation of three synthetic method of Ce-BGs.

Ce-BGs are produced by various synthetic methods, each of which corresponds to a specific Ce-BG category. The three most significant categories are discussed below and illustrated in Figure 3, starting with melt-quenching glasses (MQGs), discovered by Hench in 1969,^1^ followed by the bioactive sol–gel glasses (SGGs), also proposed by Hench in 1991,^38^ and most recently by bioactive mesoporous glasses (MBGs), designed and reported independently by Zhao and Vallet-Regi in 2004 and 2006, respectively.^39,40^

### 2.1 Melt-Quenching Glasses (MQGs)

In the melt-quenching technique, the glass precursors are melted and successively quenched; the first BG of this kind was 45S5.^1,38^ Ions belonging to the rare-earth group are used to improve the properties of BGs;^5,6^ these ions possess a high field strength and thus tend to aggregate in clusters when melted with other elements.^41,42^ The solubility of rare-earth ions in pure silicate glass is less than 1 mol % but increases in phosphate-based glass, where the formation of clusters is reduced.^41−45^

In the particular case of cerium, the easy switch between Ce^3+^ and Ce^4+^ oxidation states is the basis of its catalytic activity. The high temperatures required for melt-quenching influence the Ce^3+^/Ce^4+^ ratio, which depends also on melting isotherm, glass composition, and the partial pressure of O_2_ in the oven atmosphere.^46−48^ Moreover, a higher concentration of cerium in the glass favors the increment of Ce^4+^ concentration, while higher temperature promotes Ce^3+^ formation.^49^ At temperatures >1000 °C, in low-alkali borate and silicate glasses, the Ce^3+^ state prevails, while the Ce^4+^ is favored at higher alkali content.^50^ In sodium phosphate derived glasses, the Ce^3+^ state is favored and oxidation does not occur in the presence of oxygen, even when melting temperature reaches 1000 °C.^51^ Several papers reported that the presence of phosphate (calcium *meta*-phosphate glasses with high silica) favors the Ce^3+^ state independently of the maximum melting temperature.^52,53^

The first Ce-BGs were synthesized by Lusvardi et al. in 2002^54,55^ using CeO_2_ as the cerium precursor. The glass composition was based on 45S5 doped with different CeO_2_ amounts (1.5, 3.2, 5.3, or 13.5 wt %). Although the introduction of the rare earth decreases the viscosity of the melt,^56^ the higher amount of CeO_2_ (13.5 wt %) and its low solubility in silicate glasses required higher temperature and longer isotherm. Attempts were also made to produce glasses with higher CeO_2_ contents, which resulted in an opaque material with a clear phase separation; higher CeO_2_ content favors the monazite (CePO_4_) formation in the glass system 15SiO_2_–15Al_2_O_3_–70P_2_O_5_–(0 + *x*)CeO_2_ (*x* = 0–25 mol %).^57^

### 2.2 Sol–Gel Glasses (SGGs)

Since the 1990s, the interest toward the sol–gel synthesis has increased;^58,59^ in 1991 some BGs in ternary SiO_2_–CaO–P_2_O_5_ systems were synthesized by the sol–gel method. The main steps involved are preparation of sol, casting, gelation, aging, drying, and thermal stabilization.^5,60^ The addition of an acid catalyst (acid water-based solutions, such as HCl, HNO_3_, and CH_3_COOH^61^) during the sol–gel process is necessary in order to obtain a 3D reticulated structure. With respect to MQGs, SGGs are purer, more homogeneous in composition, and more porous and have higher specific surface area (SSA, usually ∼100–650 m^2^/g). In contrast, MQGs have no porosity, and their low SSA (∼1–2 m^2^/g) depends only on the particle size resulting from the grinding of the materials. The porosity of SGGs allows the formation of a hydrated layer inside the material, where biological moieties can enter, maintaining their structural configuration and biological activity; SGGs then can become an indistinguishable part of the host tissue. For example, it has been shown that when trabecular rabbit bone was proliferated on 45S5, large particles were still present even if a structure similar to normal bone was obtained. Conversely, no residual particles were observed when SGGs were utilized.^62^ The formation of HCA takes place much faster on the surface of SGGs than on MQGs; furthermore, HCA formation is observed in SGGs with a SiO_2_ content up to 90 wt %, while it is only observed in MQGs with 60% or less SiO_2_.^63^

The most common precursors of cerium in SGGs are Ce(NO_3_)_3_·H_2_O for Ce^3+^ and (NH_4_)_2_Ce(NO_3_)_6_ or Ce(SO_4_)_2_ for Ce^4+^.^64^ Also, for sol–gel synthesis, the equilibrium between Ce^3+^ and Ce^4+^ mainly depends on thermal treatment, glass composition, and the O_2_ partial pressure during the thermal stabilization. When a Ce^3+^ precursor is used, trivalent state normally prevails at room temperature; over 100 °C partial oxidation to Ce^4+^ starts, and from 200 to 1000 °C, cerium is completely oxidized to Ce^4+^. In the case of Ce^4+^ precursor, at room temperature a partial reduction to Ce^3+^ takes place, and over 100 °C, it tends to be reoxidized.

### 2.3 Mesoporous Glasses (MBGs)

The discovery of silica mesoporous materials (SMMs) in 1991 by company scientists of Mobil Oil Corporation was recognized as a breakthrough that could lead to a number of important applications as host–guest systems.^65,66^ SMMs are ordered porous structures of SiO_2_ that show high surface area and pore volume. The pore arrangement is regularly ordered in different geometrical shapes with narrow pore size distribution ranging from 2 to 50 nm that can be controlled and modified by using different synthetic strategies.^67^

The synthesis of MBGs is based on the sol–gel methodology, but the procedure involves the addition of a nonionic surfactant (structure directing agent, SDA) to the alcohol or aqueous solvent before the addition of oxide precursor and the subsequent evaporation-induced self-assembly (EISA) process.^68,69^

The most used SDAs are cetyltrimethylammonium bromide (CTAB), Pluronic F127, and Pluronic P123.^70,71^ In particular, cerium-containing MBGs (Ce-MBGs) were synthesized by using Pluronic P123.^72^

After solvent evaporation, the SDA concentration increases and eventually exceeds the critical micelle concentration (cmc), causing micelles to form in the solution. Subsequently, the co-self-assembly of micelle and silicate matrix leads to the formation of the mesophase. The final MBG is obtained after gelling, drying, and surfactant calcination (700 °C). The calcination of surfactant promotes a porous structure that can be ordered (mesoporous ordered structure) or not-ordered (worm-like structure), and this depends on the glass composition.

The resulting materials present a high SSA (usually ∼300–800 m^2^/g) and a significantly larger pore volume (∼1 cm^3^/g) with respect SGGs. However, the MQGs have enhanced mechanical properties like hardness and flexural strength with respect both SGGs and MBGs.^73^

MBGs exhibit higher bioactivity than SGGs due to their outstanding textural properties; moreover, MBGs can most easily incorporate species of biological importance, which can be released in controlled manner, thus acting as a drug delivery system.^74^

The formation of ordered mesoporous arrangements is regulated by factors like, among others, surfactant nature, concentration of precursors, solvent, pH, and temperature.^75−77^ In the case of SiO_2_–CaO–P_2_O_5_ system, CaO acts as a network modifier disrupting the silica network connectivity; when CaO increases, the inorganic/organic volume ratio of the micelles increases with the formation of hexagonal phases rather than cubic.^40^ P_2_O_5_ leads to a decrease in the inorganic/organic volume ratio of the micelles resulting in a cubic structure.^69,78,79^

For Ce-MBGs, the glass composition influences the Ce^3+^/Ce^4+^ ratio. The presence of P_2_O_5_ favors the Ce^3+^ state: in ternary SiO_2_–P_2_O_5_–CeO_2_ MBG calcined at 700 °C, the Ce^3+^ amount is 80.0 wt %, while in ternary SiO_2_–CaO–CeO_2_ and binary SiO_2_–CeO_2_ MBGs, the Ce^3+^ amount decreases to 37.5 and 58.0 wt %, respectively.^79^

The introduction in the glass network of cerium ions decreases the SSA and the porosity order degree;^80^ in fact it is possible to obtain a hexagonal ordered structure until 1 mol % of CeO_2_ addition, while for higher concentration, decreased SSA and a worm-like porous structure is obtained. However, it is still possible to enhance the SSA by increasing the concentration of surfactant: during the synthesis of Ce-MBGs the SSA increase around 2.5 times upon the introduction of twice the amount of surfactant (Pluronic P123).^81^

Similar results were obtained for MBGs without cerium, where the introduction of higher P123 amounts increases SSA, pore diameter, and volume.^82^

It is also possible to obtain MBGs as nanoparticles by basic catalysis (aqueous ammonia);^83,84^ the cerium-doped MBG nanoparticles are obtained by immersion in a solution of cerium nitrate after the thermal calcination at 700 °C. This process favors the exchange of Ce^3+^ ions from the solution with the Ca^2+^ ions in the glass structure; the final MBGs contain cerium ions on the glass surface.^84^

## 3 Properties of Bioactive Glasses

### 3.1 Bioactivity

In the context of synthetic bone grafts, bioactivity concerns the formation of a bond with bone. In the field of bone repair, it is more appropriately defined as a “stimulation of a beneficial biological response”.^85^ 45S5 was the first biomaterial able to bond with bone, rather than be encapsulated by fibrous tissue; the bond was so strong that could not be removed without breaking it.^1^ The mechanism of the bioactivity^1,86^ is divided into two macrostages:^87^ bone-like apatite layer formation^88^ and ionic dissolution products from BGs and osteogenesis.^4^

The general mechanism of formation of the HCA layer is well-known and thus not covered here; we focus instead on the influence of cerium on the bioactivity of Ce-BGs.

As reported in the section 2, the use of different synthetic methods modifies the SSA and the reactivity of BGs. Table 1 reports the features of the Ce-BGs studied for their bioactivity as a function of compositions, dimensions and shape (not always reported), synthetic methods, and maximum soaking time in simulated body fluid (SBF).^89^

**Table 1:** Evaluation of Bioactivity for Ce-BGs

			
composition	synthesis^a^	features (dimensions or shape, maximum time of SBF soaking)	refs
45S5 doped with CeO_2_ (0.75, 1.5, 3.2, 10, 13 wt %)	M	powders, 250–500 μm, 30 days	(54,55)
3:7 (wt %) Ca_10_(PO_4_)_6_F_2_/K_2_Mg_3_AlSi_3_O_10_F_2_ doped with CeO_2_ (1 wt %)	M	glass-ceramics, 28 days	(112)
(80 – *x*)SiO_2_–15CaO–5P_2_O_5_–*x*Ce_2_O_3_ (*x* = 0.2, 1, 2, 3.5 mol %)	SGE	MBG, pellets, Ø = 6 mm, 7 days	(72)
(80 – *x*)SiO_2_–15CaO–5P_2_O_5_–*x*Ce_2_O_3_ (*x* = 0.2, 1, 2, 3.5 mol %)	SGE	MBG, powders, <50 μm,15 days	(98)
(80 – *x*)SiO_2_–15CaO–5P_2_O_5_–*x*Ce_2_O_3_ (*x* = 0.2, 1 mol %)	SGE	MBG, scaffolds, 7 days	(97)
(80 – *x*)SiO_2_–15CaO–5P_2_O_5_–*x*Ce_2_O_3_ (*x* = 1, 2 mol %)	SGE	MBG, powders, <32 μm, 7 days	(99)
50SiO_2_–(45 – *x*)CaO–5P_2_O_5_–*x*CeO_2_ (*x* = 1, 5, 10 mol %)	SG	MBG, 14 days	(100)
*x*CeO_2_–(100 – *x*)[0.5P_2_O_5_–0.2CaO–0.2SrO–0.1Na_2_O] (*x* = 1, 2, 5, 7.5 mol %)	M	powders, 300–500 μm, 7 days	(106)
56.6B_2_O_3_–18.5CaO–5.5Na_2_O–11.1K_2_O–4.6MgO–3.7P_2_O_5_ doped with Ce_2_O_3_ (1, 3, 5 wt %)	M	scaffolds, *d*_50_ = 13.2 μm, 30 days	(113)
(53 – *x*)SiO_2_–20CaO–6Na_2_O–12K_2_O–5MgO–4P_2_O_5_–*x*Ce_2_O_3_ (*x* = 0, 1, 3, 5 wt %)	SG	electrospun fibers, Ø = 583 nm; powders, 69 < *d*_50_ < 145 μm, 30 days	(105,123)
79SiO_2_–15CaO–5P_2_O_5_–Ce_2_O_3_ (mol %)	SGE	MBG (Ø = 10 μm), 30 days	(101)
50SiO_2_–(45 – *x*)CaO–5P_2_O_5_–*x*CeO_2_ (*x* = 1, 5, 10 mol %)	SG	nanofibers (Ø = 158 nm), 7 days	(107)
52SiO_2_–24SrO–16Na_2_O–8CeO_2_ and 52SiO_2_–24SrO–16Na_2_O–4CeO_2_–4Y_2_O_3_ (mol %)	M	disks, SA 100 mm^2^, 14 days	(108)
45S5 doped with CeO_2_ (1.2, 3.6, 5.3 mol %)	M	powders, 250–500 μm, 28 days	(91,94)
K50S doped with CeO_2_ (1.2, 3.6, 5.3 mol %)			
15CaF_2_–10CaO–5B_2_O_3_–(65 – *x*)P_2_O_5_–5BaO–*x*Ce_2_O_3_ (*x* = 0, 1, 2, 3, 4 mol %)	M	slices, 1.0 cm × 1.0 cm × 2.5 cm, 30 days	(109)
70SiO_2_–(26 – *x*)CaO–4P_2_O_5_–*x*CeO_2_ (*x* = 0, 1, 5 mol %)	SGE	MBG pellets (Ø = 8 mm), 28 days	(81)
60SiO_2_–(10 – *x*)B_2_O_3_–25CaO–5P_2_O_5_–*x*CeO_2_, (*x* = 5 mol %)	SG	pellets (Ø = 8 mm), 15 days	(115)
80SiO_2_–15CaO–5P_2_O_5_ doped with CeO_2_ (5.3 mol %)	SGE	MBG powders, <250 μm, 14 days	(80)
80SiO_2_–20CaO doped with CeO_2_ (5.3 mol %)			
80SiO_2_–20P_2_O_5_ doped with CeO_2_ (5.3 mol %)			
100SiO_2_ doped with CeO_2_ (5.3 mol %)			
80SiO_2_–15CaO–5P_2_O_5_ doped with CeO_2_(1.2, 3.6, 5.3 mol %)	SGE	MBG/alginate beads: powders, <250 μm; beads, Ø = 2 mm, 28 days	(116)
20Na_2_O–14CaO–*x*CeO_2_–(66 – *x*)P_2_O_5_ (*x* = 0.1, 0.3, 0.7, 1 wt %)	M	cubic shape, 21 days	(110)
K50S doped with of CeO_2_ (1.2, 3.6, 5.3 mol %)	M	slices (thickness = 3 mm, surface area = 1 cm^2^), 30 days	(96)
34SiO_2_–8P_2_O_5_–17MgO–*x*CeO_2_·(41 – *x*)CaO (*x* = 0.5, 2.5, 5 mol %)	SG	powders, 130–190 nm, 14 days	(111)
45S5 doped with CeO_2_ (4, 5 mol %)	M, SGE	45S5, K50S, MBG/alginate beads: powders <250 μm; beads Ø = 2 mm, 28 days	(92)
K50S doped with CeO_2_ (3.6 mol %)			
80SiO_2_–15CaO–5P_2_O_5_ doped with CeO_2_ (5.3 mol %)			
(45 – *x*)SiO_2_–24.5Na_2_O–24.5CaO–6P_2_O_5_ (*x* = 0.5, 1, 1.5, 2 wt %) equal amount of CeO_2_ and La_2_O_3_	M	powders <60 μm, 19 days	(93)

a M = melt-quenching; SG = sol–gel; SGE = sol–gel EISA.

*In vitro* studies have been carried out on BGs of different types, namely, 45S5,^55,90−94^ Kokubo glass (N25C25S50, hereafter abbreviated as K50S),^92,95,96^ MBGs,^74,81,85,93,97−102^ 13-93,^103−105^ and other BGs^106−111^ doped with different amounts of cerium and synthesized by melt-quenching,^54,55,91−93,96,106,108−110,112,113^ sol–gel,^100,103,105,107,111,114,115^ and sol–gel EISA^72,80,81,97−99,101,102,116^ methods.

**Figure 4:**
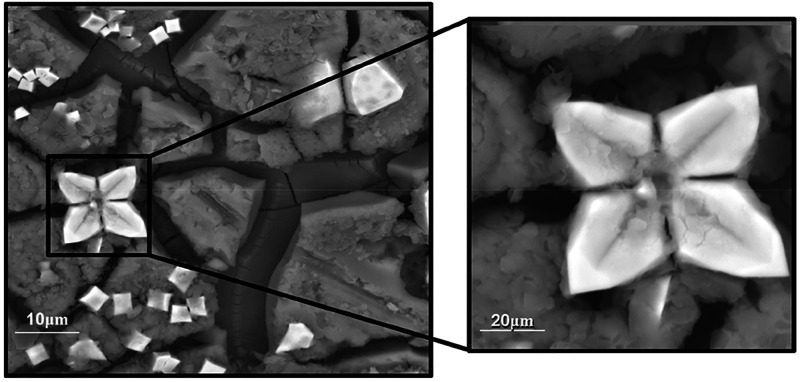
SEM micrographs BG-13 Ce glass after 30 days of soaking in SBF Reproduced with permission from ref (55). Copyright 2003 Elsevier.

The first comments are related to Ce-MQGs in Lusvardi et al.^55^ where the cerium content was first reported as improving the chemical durability and retarding the HCA layer formation, mainly due to two factors: (i) the increase in chemical durability and (ii) the formation of insoluble crystalline CePO_4_, competitive with HCA. CePO_4_ is very insoluble (Ksp_CePO4_ = 10^–23^),^117,118^ and this hampers further solubilization of the glass matrix. This effect is correlated with the CeO_2_ amount in the glass: for CeO_2_ content up to 1 mol %, HCA formation was detectable after 7–14 days, while with higher CeO_2_ content (5.3 mol %), the formation of HCA was delayed up to 28 days.^92^ In this study, the formation of insoluble Ce-phosphate phase was detected by SEM analysis, with typical flower-like crystals on the glass surface after SBF soaking (Figure 4).

Subsequently, a similar behavior was detected for SGGs and MBGs.^100,103,105,107,111,114,115^

In particular, MBGs containing up to 5.3 mol % CeO_2_ showed HCA after 7–14 days of SBF soaking.^116^ Here, the simultaneous presence of both HCA and CePO_4_ confirmed that the Ce^3+^ ions released by the glass surface react quickly with the phosphate ions of the SBF forming the CePO_4_ insoluble phase. This also explains the low level of cerium ions in SBF^72^ (cerium concentration <0.05 ppm). In summary, the presence of cerium does not inhibit HCA formation but can delay it at high concentrations due to the competitive cerium phosphate phase, sometimes identified as CePO_4_.^70,71,82−84,86,87,102^

In order to manufacture BGs with higher bioactivity, a suitable morphology can be selected. Some authors^38,116,119,120^ used Ce-MBGs as a bioactive filler in alginate beads to increase their bioactivity and pro-osteogenic activity. The results indicate that beads with 1.2 and 3.6 mol % CeO_2_ are excellent candidates as biocompatible scaffolds.

A final general consideration concerns SBF tests. Direct comparison of literature data on the HCA layer formation is often problematic as the protocols used for SBF testing can vary between research groups. The ISO standard currently in use^121^ refers to materials of standard shape but does not take into account that BGs can have very different specific surface areas and the required amount of SBF should be appropriately chosen. A unified assessment method based on an ISO modified procedure, considering the ratio between BG mass and SBF solution, has been recently proposed.^122^

### 3.2 Cytocompatibilty

Cell culture methods are the main *in vitro* tool to predict the biological response of the host organism to a biomaterial (Table 2). The cell lines selected for these assays are then typically chosen to model the response likely observed *in vivo* upon the surgical implant of BGs.^122,124^ Accordingly, the cell types commonly employed to assess the cytocompatibility of BGs have a role in wound healing (fibroblasts),^81,93,115,125,126^ bone structure (osteocytes),^96,127,128^ and bone maintenance and formation (osteoclasts and osteoblasts).^108,111,116,129^ As cell cultures are sensitive to changes in variables such as temperature, pH, and nutrient concentration, careful control of the experimental conditions is crucial in correlating cell death to toxicity of the biomaterial rather than to changes in the culture conditions.^122^ The assessment of cellular response to BGs, and their cytotoxicity in particular is performed by direct tests, carried out in the presence of the BGs, and indirect ones, in which filtered extracts of BGs are added to the cell culture.^130^ Among the latter, 3-(4,5-dimethylthiazol-2-yl)-2,5-diphenyltetrazolium bromide test (MTT) is the method of choice for the quantification of metabolically active cells upon incubation with BG eluates.^81,96,108,125,127,131^ MTT is a rapid colorimetric test based on the cleavage of a yellow tetrazolium salt to purple formazan crystals by mitochondrial enzymes in metabolically active cells.^122^ Another indirect assay reported on BG extracts is the alamarBlue assay for cell viability (applicable both as a direct and indirect test).^116^ All the BGs studied show excellent biocompatibility regardless of their cerium content^81,96,108,116,125−127^ with specific exceptions for reused materials.^127^ Lactate dehydrogenase (LDH) activity is also used to assess the cytotoxicity of BGs in indirect assays;^81,116^ both studies show no significant difference between control medium and extracts, confirming the lack of cytotoxicity of BGs.

**Table 2:** Evaluation of Cytocompatibility for Ce-BGs

composition	assays and cell lines	features	ref
10CaF_2_–10Na_2_CO_3_–15CaO-60P_2_O_5_–5CeO_2_ (mol %)	MTT, human osteoblastic-like cells MG63 cells (ATCC, CRL-1427)	enhanced cell adhesion and proliferation	(129)
52SiO_2_–24SrO–16Na_2_O–8CeO_2_ (mol %)	osteoblast viability, cell adhesion MC-3T3-E1 osteoblasts (ATCC CRL-2593)	cell viability unaffected	(108)
52SiO_2_–24SrO–16Na_2_O–4CeO_2_–4Y_2_O_3_ (mol %)			
70SiO_2_–(26 – *x*)CaO–4P_2_O_5_–*x*CeO_2_ (*x* = 1, 5 mol %)	MTT, LDH, mouse fibroblast cells (NCTC clone L929)	cell viability above 80% noncytotoxic	(81)
60SiO_2_–(10 – *x*)B_2_O_3_–25CaO–5P_2_O_5_–*x*CeO_2_, (*x* = 0, 5 mol %)	MTT	cell viability improved	(115)
	human lung fibroblast normal cells (WI-38 cells)		
80SiO_2_–15CaO–5P_2_O_5_ (mol %) doped with CeO_2_ (1.2, 3.6, 5.3 mol %)	MTT, LDH, ALP	cell viability unaffected, cerium enhances cells proliferation and reduces cell differentiation	(116)
	mouse calvaria preosteoblastic cells (MC3T3-E1)		
45S5 doped with CeO_2_ (1.2, 3.6, 5.3 mol %)	NR, XTT, BRdU, MLO-Y4, NIH/3T3 cell lines	cell uptake and viability enhanced	(128)
		cerium enhances cells proliferation	
K50S doped with CeO_2_ (1.2, 3.6, 5.3 mol %)	MTT, NR, BrDU	cell proliferation and vitality enhanced	(96)
	osteocyte-like cell lines murine long bone (MLO-Y4)		
34SiO_2_–8P_2_O_5_–17MgO–*x*CeO_2_–(41 – *x*)CaO (*x* = 0.5, 2.5, 5 mol %)	MTT	cerium reduces apoptosis and increases cell viability	(111)
	human osteosarcoma cells (MG-63)		
(45 – *x*)–SiO_2_–24.5Na_2_O–24.5CaO–6P_2_O_5_ (wt %) *x* = 0.5,1.0, 1.5, 2.0, equal wt % of CeO_2_ and La_2_O_3_	MTT	cerium reduces apoptosis and increases cell proliferation	(93)
	mouse fibroblast L929 cell lines		
45S5 doped with CeO_2_ (1.2, 3.6, 5.3 mol %)	NR, MTT, BRdU	cell uptake and viability enhanced	(127)
	MLO-Y4, NIH/3T3 cell lines	cell proliferation reduced in the second use	
46.10SiO_2_–2.60P_2_O_5_–16.90CaO–10.00MgO–19.40Na_2_O–5.00CeO_2_(mol %)	MTT, human fibroblast BJ cells (ATCC, CRL-2522)	cell viability around 90%	(125)
60SiO_2_–28CaO–4P_2_O_5_–8Ce_2_O_3_ (mol %)	MTT, NIH 3T3 mouse fibroblast cells	cerium enhances cell adhesion and spreading	(126)
70SiO_2_–30CaO impregnated (Ce 0.05, 0.2 M)	MTT	no cytotoxic	(83)
		cerium reduces expression of oxidative stress related genes	
34SiO_2_–P_2_O_5_–17MgO–*x*CeO_2_–(41–*x*)CaO	MTT, osteoblasts (rats)	cerium enhances cell vialability	(111)

Direct cytocompatibility has been assessed by a range of assays, including MTT,^83,93,111,115,125,126,129,131^ alamarBlue,^116^ and neutral red (NR)^96,127,128^ for cell viability, bromo-2-deoxyuridine (BrdU) for cell proliferation,^96,127^ and LDH activity for cytotoxicity.^81,116^ Remarkably, all the BGs investigated show little^93,115^ or no^83,96,111,126,127,129^ effect on cell viability and are cytotoxic regardless of the amount of cerium in the glass composition;^81,116,128^ in some instances, the amount of cerium in the glass composition increases the biocompatibility of the materials.^93,115,128,129^ At very long (14 days) culture times, cell viability is reported to decrease, and BGs with a higher amount of cerium show better cytocompatibility.^126^ Conversely, when H_2_O_2_ is added to the culture medium to simulate conditions of oxidative stress, the presence of cerium has a marked positive effect on cell viability, consistent with the antioxidant properties of Ce-BGs.^111,116^ Specific assays are also used to study hemolysis,^93^ cell apoptosis,^93,111^ and alkaline phosphatase (ALP) as a marker of osteoblast activity.^116,129^ Ce-BGs induced lower hemolysis^93^ and apoptosis^111^ than nondoped BGs, while decreasing ALP activity, as to be expected by their cell proliferation effect.^116^ Recently it has been also demonstrated that the incorporation of cerium into mesoporous bioactive glass nanoparticles (MBGNs) reduces the expression of oxidative stress-related genes in macrophages (J774a.1).^83^

Finally, SEM or confocal microscopy are used to evaluate changes in cell morphology and cell adhesion to the surface of the BGs. The cell morphology is generally unchanged upon interaction with BGs^96,116,125,126,128^ if not at higher BG concentration;^115^ the presence of cerium reduces morphological changes^115^ and gives better performance over unfunctionalized BGs.^96,126^ Cell attachment is also favored by the presence of cerium.^108,129^

### 3.3 Antibacterial Activity

The efficiency of BGs in bone regeneration is also related to the prevention of bacterial adhesion and proliferation that can occur on the implant surface. While BGs are considered good candidates for preventing or reducing this problem,^133^ the mechanisms underlying their antibacterial activity are still under study. Among the reported modes of action are the disruption of prokaryotic cell membranes by glass debris^134,135^ and changes in environmental pH and osmotic pressure.^136^ Both those mechanisms are linked to the reactivity of BGs in aqueous solutions, with produces a toxic environment for bacteria. This behavior is associated with an increase of pH and osmolarity in the surrounding environment; an alkaline pH reduces the viability of bacterial suspensions and causes morphological and ultrastructural changes in the bacteria.^137^ The antibacterial properties can also be induced or improved by the addition of metal ions with bactericidal effects. BGs doped with silver, copper, zinc, or gallium are considered potential candidates as antibacterial agents.^4,5^ Ce-BGs as well are reported as having antibacterial properties, with microbicidal effects toward *Escherichia coli*(100,110,125) and *Staphylococcus aureus*(129) (Table 3), albeit some studies report the lack of such properties instead.^5,93,94,97^ The antibacterial activity of cerium compounds is linked to the inhibition of the oxidation and assimilation of glucose and of endogenous respiration.^138^ Various modes of action of cerium compounds on bacteria have been proposed, some of which are based on the direct contact between cerium and the bacterial membrane.^139^ These include the impairment of transport exchange through the bacterial membrane followed by reduced growth,^110,140^ reaction of cerium with proteins or transporters within the cell,^135^ and induction of oxidative stress.^141,142^

**Table 3:** Evaluation of Antibacterial Activity for Ce-BGs

composition	bacterial strain	antibacterial effect	ref
50SiO_2_–(45 – *x*)CaO–5P_2_O_5_–*x*CeO_2_(*x* = 1, 5, 10 mol %)	*E. coli* (ATCC25922)	increasing the amount of cerium increases the antibacterial activity	(100)
10CaF_2_–10Na_2_CO_3_–15CaO-60P_2_O_5_–5CeO_2_ (mol %)	*S. aureus* (ATCC 25923)	antibacterial effect against *S. aureus* and *S. epidermidis*	(129)
	*S. epidermidis* (ATCC 35984/RP62A)		
	*P. aeruginosa*	no effects against *P. aeruginosa*	
56.6B_2_O_3_–18.5CaO–5.5Na_2_O–11.1K_2_O–4.6MgO–3.7P_2_O_5_ doped with Ce_2_O_3_ (1, 3, 5 wt %)	*E. coli*	no antibacterial response	(123)
	*S. aureus*		
(53 – *x*)SiO_2_–20CaO–6Na_2_O–K_2_O–MgO–P_2_O_5_–*x*Ce_2_O_3_ (*x* = 3, 5 wt %)	*S. aureus* (ATCC25923)	no antibacterial response	(105)
	*E. coli* (ATCC25922)		
50SiO_2_–(45 – *x*)CaO–5P_2_O_5_–*x*CeO_2_ (*x* = 1, 5, 10 mol %)	*E. coli* (ATCC25922)	no antibacterial response	(107)
60SiO_2_–(10 – *x*)B_2_O_3_–25CaO–5P_2_O_5_–*x*CeO_2_(*x* = 5 mol %)	*E. coli* (ATCC 25922)	antibacterial activity did not depend on cerium presence	(115)
	*P. aeruginosa* (ATCC 27853)		
	*Bacillus subtilis* (ATCC 6633)		
	*S. aureus* (ATCC 6538)		
20Na_2_O–14CaO–*x*CeO_2_–(66 – *x*)P_2_O_5_ (*x* = 0.1, 0.3, 0.7, 1 wt %)	*S. aureus*	antibacterial activity enhanced significantly against *E. coli* and *S. aureus* as cerium amount increases	(110)
	*B. cereus*	no antimicrobial behavior against *B. cereus, B. subtilis*, and *C. albicans*	
	*B. subtilis*		
	*E. coli*		
	*C. albicans*		
46.1SiO_2_–2.6P_2_O_5_–16.9CaO–10.0MgO–19.4Na_2_O–5.0CeO_2_ (mol %)	*E. coli* (K12-MG1655)	high antibacterial activity for coatings obtained by PLD	(125)

More recent studies, performed between 2014 and 2020, suggest that the antibacterial activity of Ce-BGs is a function of glass composition, cerium amount, and morphology.

Ce-BGs possess higher antibacterial activity if the concentration of cerium oxide is in the 5–10 mol % range rather than 1 mol %.^100^ Ce-BG-reinforced hydroxyapatite showed a remarkable decrease of bacterial adhesion only for the *Staphylococcus* strains.^129^ Electrospun fibers and powders based on 13-93 glasses doped with cerium^105,123^ and electrospun poly(lactic acid) (PLA)/chitosan nanofibers coated with cerium-doped glasses are inactive in antibacterial tests; this lack of antibacterial activity can be attributed to the slow release of ions from glass and to the small amount of material adsorbed onto the nanofibers.^107^ The antibacterial activity of Ce-nano-BGs is not dependent on the presence of cerium in the glass but rather on the presence of boron, which shows antibacterial activity against a wide range of pathogens.^115^ For cerium-containing phosphate glasses, the increase of cerium concentration enhanced the antibacterial activity against *E. coli* and *S. aureus*, but not against *Bacillus cereus*, *B. subtilis*, and *Candida albicans*.^110^

Preliminary tests performed on coatings obtained by the laser ablation method and enriched with Ce-BGs suggested high antibacterial activity due to the presence of partially crystallized layers with cerium cations embedded in a glassy matrix, which was more prone to degradation.^125^

### 3.4 Antioxidant Properties

Oxidative stress is related to the excessive production of ROS, and these species play an important role in the regulation of cellular functions: inhibition of the differentiation and mineralization of osteoblasts, enhancement of osteoclast activity, and consequent pro-inflammatory bone resorption.^143^ Their excess can have deleterious effects on the organism with a reduction of antioxidant capacity.^144^ The implantation of biomedical devices is performed by surgical procedures, which are often followed by tissue damage and inflammation. ROS production linked to inflammation increases and causes a condition of oxidative stress, which in turn enhances inflammation, causing further generation of ROS. Due to this feedback, postsurgery inflammation could need a long time to achieve complete recovery.

The ability to convert ROS to nondangerous species must be a key feature of a biomaterial.

In the case of nanoparticles, CeNPs have been widely studied for their antioxidant enzyme-mimetic activity and radical scavenging ability.^19,20^ In the site of the inflammation, CeNPs favor the conversion of excess free radicals, bringing a faster postsurgery recovery;^145^ their antioxidant properties are effective against ROS generated in the human body.^18^ CeNPs can mimic the activity of catalase (CAT)^146^ and superoxide dismutase (SOD)^147^ enzymes present in the human body^147^ (Figure 2).

The antioxidant properties of BGs are strictly correlated to their composition and reactivity. For example, the addition of fluorine (5–15 mol %) to 45S5 increases lipid peroxidation and ROS production in MG-63 osteoblast cells and induces other signs of oxidative stress such as inhibition of the pentose phosphate pathway, the glucose 6-phosphate dehydrogenase activity, and the glutathione activity.^148^ Similarly, the introduction of copper (1–2.5 wt %) into 45S5 increases ROS production in human osteosarcoma (HOS) cells.^149^

Table 4 summarizes the results related to Ce-BGs and their potential antioxidant activity.

**Table 4:** Evaluation of Antioxidant Activity for Ce-BGs

composition	antioxidant activity	features	ref.
45S5 doped with CeO_2_ (1.2, 3.6, 5.3 mol %)	CMA	antioxidant activity increases with the increase of cerium amount, decreases in presence of phosphate ions, and changes with the environment (higher in water than in SBF)	(91, 94, 150−152)
K50S doped with CeO_2_ (1.2, 3.6, 5.3 mol %)	SOD		
80SiO_2_–15CaO–5P_2_O_5_ doped with CeO_2_ (5.3 mol %)	CMA	antioxidant activity decreases with high P_2_O_5_ amount	(80)
80SiO_2_–20CaO doped with CeO_2_ (5.3 mol %)	SOD	Ce^3+^/Ce^4+^ ratio opposite effects for CMA and SOD	
80SiO_2_–20P_2_O_5_ doped with CeO_2_ (5.3 mol %)			
100SiO_2_ doped with CeO_2_ (5.3 mol %)			
80SiO_2_–15CaO–5P_2_O_5_ doped with CeO_2_ (1.2, 3.6, 5.3 mol %)	CMA	antioxidant activity increases with the increase of cerium amount	(116)
	SOD	alginate matrix does not influence antioxidant activity	
34SiO_2_–8P_2_O_5_–17MgO–*x*CeO_2_–(41 – *x*)CaO (*x* = 0.5, 2.5, 5 mol %)	oxidative stress induced by H_2_O_2_ on MG-63 cells	cerium-containing glasses exhibit maximum cell viability	(111)
45S5 doped with CeO_2_ (4, 5 mol %)	CMA	CMA increases with (i) reduction of glass dimensions and (ii) increment of SSA; alginate coating (beads) does not inhibit CMA	(92)
K50S doped with CeO_2_ (3.6 mol %)			
80SiO_2_–15CaO–5P_2_O_5_ doped with CeO_2_ (5.3 mol %)			

CeO_2_ (1.2, 3.6, 5.3 mol %) has been added to 45S5 and K50S;^91,94,150−152^ CAT, evaluated by H_2_O_2_ degradation, increases with cerium content and decreases in the presence of phosphate groups. Cerium ions play different structural roles: in phosphate-free glasses cerium is coordinated by nonbridging oxygens (NBOs) originating from the disruption of the silicate network, whereas in phosphate-containing glasses, the NBOs around cerium ions belong to orthophosphate groups. The latter groups stabilize the Ce^3+^ species subtracting them from the interconversion process between Ce^3+^ and Ce^4+^, which is of fundamental importance for CAT. Good catalytic activities were confirmed from SOD mimic activity tests.^153^ An increase in the cerium content also leads to a significant reduction of the glass *in vitro* bioactivity, which can be associated with the formation of an insoluble CePO_4_ phase^82^ that delays or inhibits HCA formation. An optimal compromise between the ability to degrade H_2_O_2_ and HCA formation was observed with addition of 1.2 and 3.6 mol % CeO_2_.

In the case of Ce-MBGs, good bioactivity and antioxidant properties were confirmed.^80,116^ In analogy to what was observed for other BGs, the presence of a high concentration of phosphate groups decreased the catalytic properties.

Similarly to CeNPs, also in the case of Ce-BGs the Ce^3+^/Ce^4+^ ratio influences the catalytic properties. For Ce-MQGs, during CAT tests, the Ce^3+/^Ce^4+^ ratios reached an optimal value around 1–1.5. In the case of Ce-SGBs and Ce-MBGs more oxidized surfaces show improved CAT and lower SOD mimetic activity. CAT increases with smaller dimensions of the BGs and with SSA; while alginate coating (beads) seems to not inhibit the catalytic activity of the glass.^92^ CAT changes also with the environment, being higher in water than in SBF.^91,94,150−152^

### 3.5 Osteogenesis and Angiogenesis

**Figure 5:**
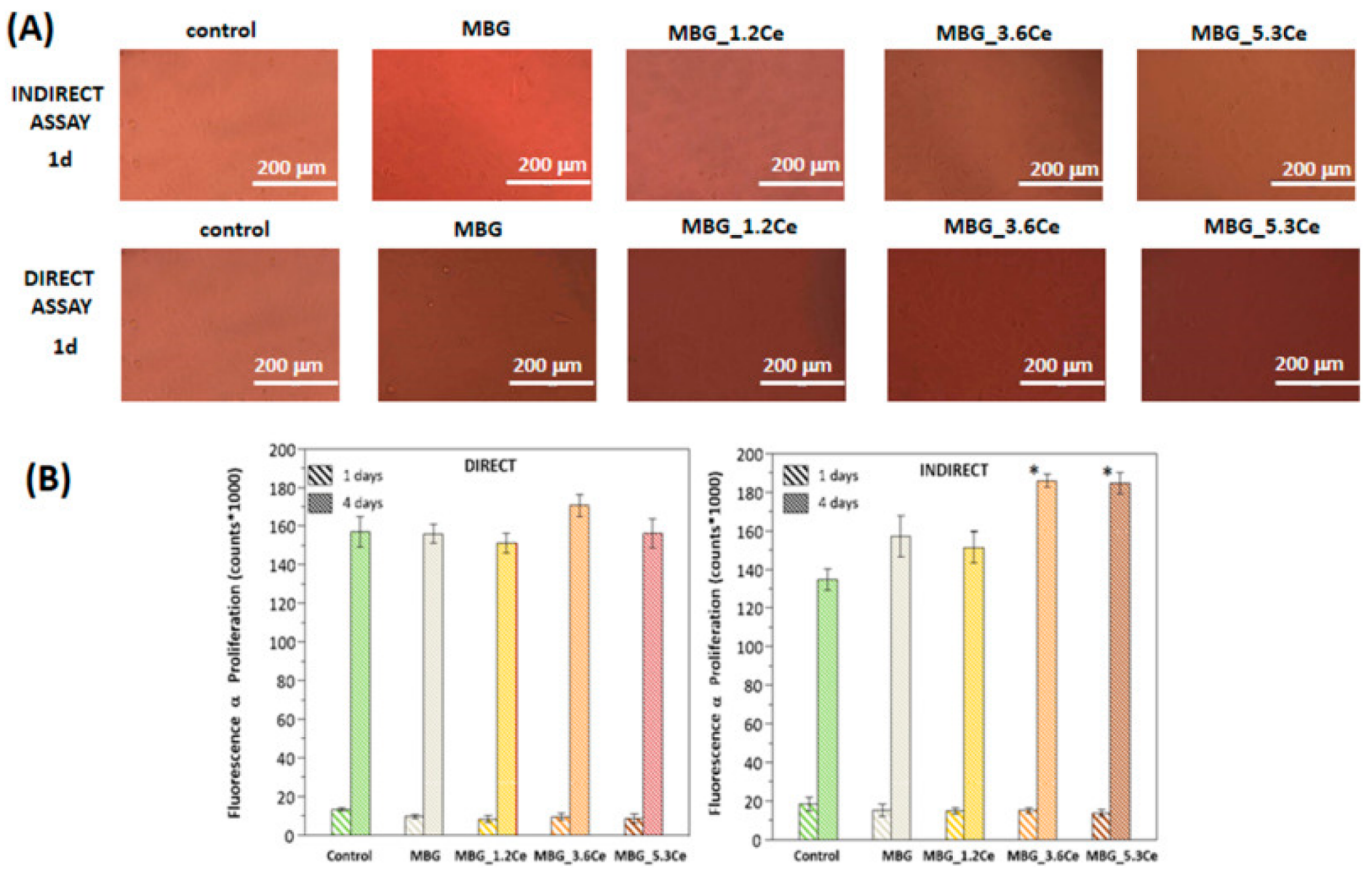
(A) Morphological evaluation of cell viability of preosteoblast cells after 1 d of culture using indirect and direct assays. (B) Cell viability (Alamar Blue) of preosteoblastic cells after 1 and 4 d of culture. (* = significant differences between control and samples after 4 d, *p* < 0.05). Reproduced with permission from ref (116). Copyright 2019 Elsevier.

BGs are also known in the field of tissue engineering because of their osteoinductivity and osteoconductivity, which are higher than those of conventional ceramics.^154,155^ TIIs, including cerium, have been added do BGs to improve their biological properties.^2,6^ The osteogenic properties of cerium compounds and CeNPs are well-known^5,156^ and linked to the ability of cerium to activate specific cellular pathways such as tumor necrosis factor (TNF)^11^ and sucrose nonfermentable (SNF).^10^ Ce-MBGs^116^ are used as bioactive filler in alginate beads to increase bioactivity and pro-osteogenic activities (Figure 5).

**Figure 6:**
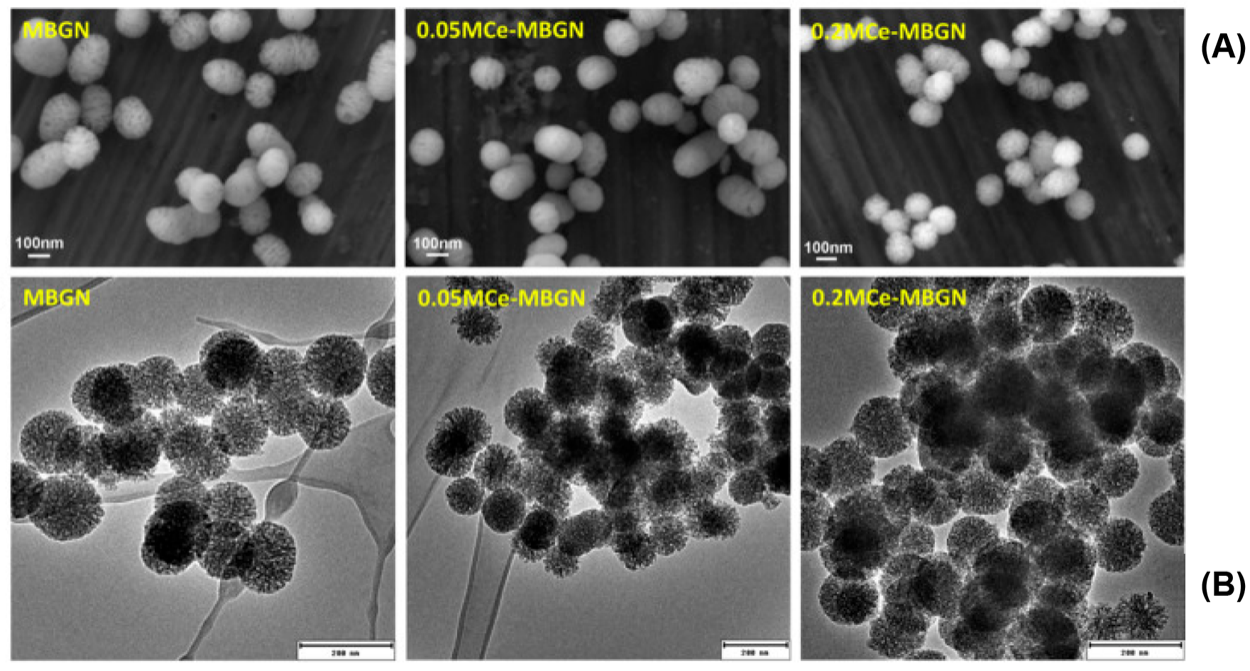
(A) SEM and (B) TEM images of the morphology of MBGN, 0.05 M Ce–MBGN, and 0.2 M Ce–MBGN. MBGN, mesoporous bioactive glass nanoparticle. Reproduced with permission from ref (83). Copyright 2020 Elsevier.

Zheng et al.^83,84,157^ incorporated cerium into MBGNPs by a two-step approach via post modification method: the nanoparticles exhibited anti-inflammatory response and pro-osteogenic activity (Figure 6)

Most of the studies on Ce-BGs for application in bone tissue regeneration report positive results with regard to osteogenic properties.^9,81,83,104^ Recently Westhauser et al.^84^ demonstrated that in MBGNs cerium had a positive influence on the viability and the cellular osteogenic differentiation of human bone marrow derived mesenchymal stromal cells exposed to the ionic dissolution products (IDPs) of the respective glasses. The formation and calcification of the osseous extracellular matrix was stimulated in the presence of IDPs of Ce-MBGNs in a positive concentration dependent manner.

Regarding angiogenesis, cerium oxide could improve the vascularization of bone grafts by activating the calcium channel of mesenchymal stem cells.^158^ Ce-BGs can modulate the oxygen level in vitro, suggesting their angiogenic potential.^91^ Ce doped borate BGs exhibited enhanced *in vivo* blood vessel formation, which was considered to be due to the presence of cerium.^104^

**Figure 7:**
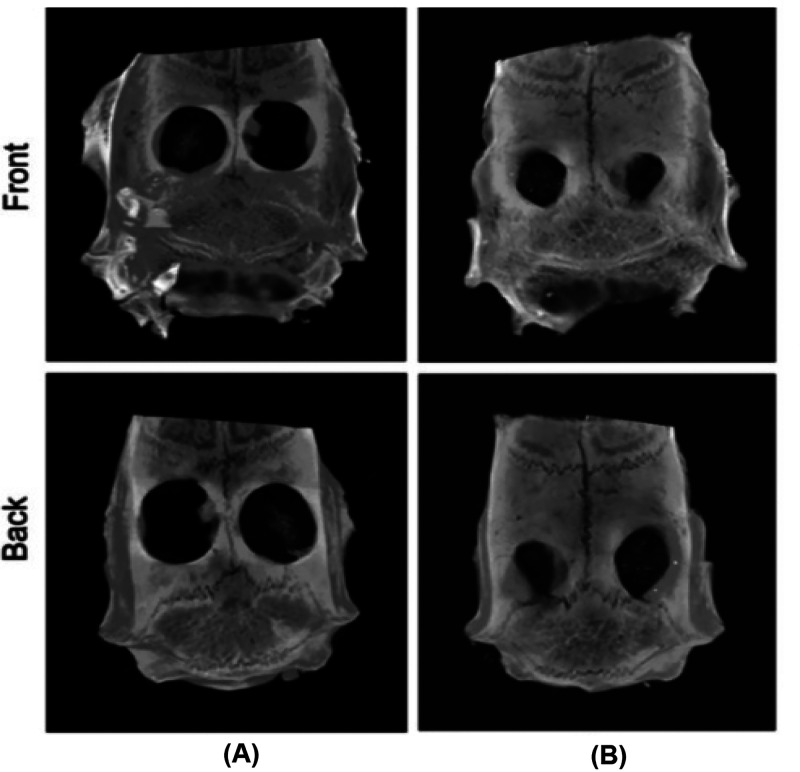
*In vivo* evaluation of bone formation in rat cranial defects implanted with BG (A) and (B) Ce-BG at 8 weeks postimplantation. The reconstruction images of micro-CT in defect regions. Reproduced with permission from ref (159). Copyright 2019 IOPScience.

*In vivo* studies on rat cranial defect models revealed that hollow mesoporous Ce-BG scaffolds accelerated collagen deposition, osteoblast formation, and bone regeneration as compared to BG scaffolds (Figure 7); these results indicate these scaffolds a promising platform for healing critical-sized bone defects.^159^

## 4 Conclusions

BGs are able to stimulate bone regeneration and are used as bone fillers, scaffolds, and implant coatings. To improve their biocompatibility and reduce postimplantation complications, BGs are doped with TIIs; among these, cerium is of particularly interest due to its biological properties. The purpose of this review is to provide an overview of the state of the art of Ce-BGs by reviewing the effects of cerium on bioactivity, cytocompatibility, and antibacterial, antioxidant, osteogenic, and angiogenic activities of BGs reported in the recent literature.

In order to explain the behavior of a Ce-BG in a biological setting, it is necessary to take into account all the manufacturing and physicochemical parameters that can influence its behavior. For instance, Ce-BG reactivity changes according to the method of synthesis described in section 2, with SGGs being more reactive than MQGs and MBGs being the most reactive glass type. We propose that a correct evaluation of the bioactivity should be performed according to the updated ISO standard and moreover that the bioactivity should be evaluated considering the composition, synthesis, and soaking time in SBF of the material (Table 1). Cytocompatibility and antibacterial and antioxidant activities are reported as a function of the composition with the most important remarks (Tables 2, 3, and 4). While in general the addition of cerium does not alter significantly the *in vitro* bioactivity of Ce-BGs, except when added in large amounts, it has a positive effect on their biocompatibility, improving their cytotoxicity and antioxidant and antibacterial properties.

Recently, Ce-BGs were also reported to have significant osteogenic properties and to help bone tissue regeneration, while Ce-doped borate BGs exhibited enhanced *in vivo* blood vessel formation, showcasing the potential benefits of these materials for a range of therapeutic areas.

In comparison with CeNPs, we can say first that CeNPs can have a large range of biomedical applications; even if it is worth considering that their employment, as with all NPs, is quite recent when compared to more established materials like the BGs that have been used for decades in tissue engineering. In addition, the compositional limitations of CeNPs reduce their versatility compared to traditional biomaterials, and the risk of cytotoxicity may be a hurdle for their approval for clinical use and subsequent commercialization.

In summary, the past decade has seen significant progresses in the application of Ce-BGs for therapeutics. Their field of application has broadened considerably and is not limited to the reconstruction of hard tissues such as bone and teeth. Ce-BGs are now explored as therapeutic options for soft tissue and are promising for adding antioxidant, antibacterial, osteogenic, and angiogenic properties.

## References

[ref1] Hench L. L.; Splinter R. J.; Allen W. C.; Greenlee T. K. Bonding Mechanisms at the Interface of Ceramic Prosthetic Materials. J. Biomed. Mater. Res. 1971, 5 (6), 117–141. 10.1002/jbm.820050611.

[ref2] Hoppe A.; Güldal N. S.; Boccaccini A. R. A Review of the Biological Response to Ionic Dissolution Products from Bioactive Glasses and Glass-Ceramics. Biomaterials 2011, 32, 2757–2774. 10.1016/j.biomaterials.2011.01.004. 21292319

[ref3] Hench L. L. J. Am. Ceram. Soc. 1998, 81, 1705–1728. 10.1111/j.1151-2916.1998.tb02540.x.

[ref4] Jones J. R. Review of Bioactive Glass: From Hench to Hybrids. Acta Biomater. 2013, 9 (1), 4457–4486. 10.1016/j.actbio.2012.08.023. 22922331

[ref5] Mehrabi T.; Mesgar A. S.; Mohammadi Z. Bioactive Glasses: A Promising Therapeutic Ion Release Strategy for Enhancing Wound Healing. ACS Biomater. Sci. Eng. 2020, 6 (10), 5399–5430. 10.1021/acsbiomaterials.0c00528. 33320556

[ref6] Kaya S.; Cresswell M.; Boccaccini A. R. Mesoporous Silica-Based Bioactive Glasses for Antibiotic-Free Antibacterial Applications. Mater. Sci. Eng., C 2018, 83, 99–107. 10.1016/j.msec.2017.11.003. 29208293

[ref7] Hoppe A.; Mouriño V.; Boccaccini A. R. Biomaterials Science MINIREVIEW Therapeutic Inorganic Ions in Bioactive Glasses to Enhance Bone Formation and Beyond. Biomater. Sci. 2013, 1 (1), 254–256. 10.1039/C2BM00116K. 32481850

[ref8] Mouriño V.; Vidotto R.; Cattalini J. P.; Boccaccini A. R. Enhancing Biological Activity of Bioactive Glass Scaffolds by Inorganic Ion Delivery for Bone Tissue Engineering. Curr. Opin. Biomed. Eng. 2019, 10, 23–34. 10.1016/j.cobme.2019.02.002.

[ref9] Zhang J.; Liu C.; Li Y.; Sun J.; Wang P.; Di K.; Zhao Y. Effect of Cerium Ion on the Proliferation, Differentiation and Mineralization Function of Primary Mouse Osteoblasts in Vitro. J. Rare Earths 2010, 28 (1), 138–142. 10.1016/S1002-0721(09)60067-3.

[ref10] Hu Y.; Du Y.; Jiang H.; Jiang G.-S. Cerium Promotes Bone Marrow Stromal Cells Migration and Osteogenic Differentiation via Smad1/5/8 Signaling Pathway. Int. J. Clin. Exp. Pathol. 2014, 7 (8), 5369–5378. 25197425 PMC4152115

[ref11] Liu D.-D.; Zhang J.-C.; Zhang Q.; Wang S.-X.; Yang M.-S. TGF-b/BMP Signaling Pathway Is Involved in Cerium-Promoted Osteogenic Differentiation of Mesenchymal Stem Cells. J. Cell. Biochem. 2013, 114, 1105 10.1002/jcb.24451. 23150386

[ref12] Jakupec M. A.; Unfried P.; Keppler B. K. Pharmacological Properties of Cerium Compunds. Reviews of Physiology Biochemistry and Pharmacology 2005, 153, 101–111. 10.1007/s10254-004-0024-6. 15674649

[ref13] Gordh T.; Rydin H. The Question of Cerium Oxalate as a Prophylactic against Postoperative Vomiting. Anesthesiology 1946, 7, 526–535. 10.1097/00000542-194609000-00006. 20996697

[ref14] Biba F.; Groessl M.; Egger A.; Jakupec M. A.; Keppler B. K. A Novel Cytotoxic Cerium Complex: Aquatrichloridobis(1,10-Phenanthroline)Cerium(III) (KP776). Synthesis, Characterization, Behavior in H_2_O, Binding towards Biomolecules, and Antiproliferative Activity. Chem. Biodiversity 2009, 6 (12), 2153–2165. 10.1002/cbdv.200900011. 20020447

[ref15] Wason M. S.; Zhao J. Cerium Oxide Nanoparticles: Potential Applications for Cancer and Other Diseases. Am. J. Transl. Res. 2013, 5 (2), 126–131. 23573358 PMC3612509

[ref16] Xu C.; Qu X. Cerium Oxide Nanoparticle: A Remarkably Versatile Rare Earth Nanomaterial for Biological Applications. NPG Asia Mater. 2014, 6 (3), e90 10.1038/am.2013.88.

[ref17] Kargozar S.; Baino F.; Hoseini S. J.; Hamzehlou S.; Darroudi M.; Verdi J.; Hasanzadeh L.; Kim H.-W.; Mozafari M. Biomedical Applications of Nanoceria: New Roles for an Old Player. Nanomedicine (London, U. K.) 2018, 13 (23), 3051–3069. 10.2217/nnm-2018-0189. 30507347

[ref18] Celardo I.; Pedersen J. Z.; Traversa E.; Ghibelli L. Pharmacological Potential of Cerium Oxide Nanoparticles. Nanoscale 2011, 3 (4), 1411–1420. 10.1039/c0nr00875c. 21369578

[ref19] Pagano G. Rare Earth Elements in Human and Environmental Health; Jenny Stanford Publishing, 2016; 10.1201/9781315364735.

[ref20] Nelson B. C.; Johnson M. E.; Walker M. L.; Riley K. R.; Sims C. M. Antioxidant Cerium Oxide Nanoparticles in Biology and Medicine. Antioxidants 2016, 5, 15 10.3390/antiox5020015. PMC4931536 27196936

[ref21] Mala R.; Ruby Celsia A. S. Toxicity of Nanomaterials to Biomedical Applications- A Review. In Fundamental Biomaterials: Ceramics; Thomas S., Balakrishnan P., Sreekala M. S., Eds.; Woodhead Publishing Series in Biomaterials; Woodhead Publishing, 2018; Chapter 15, pp 439–473, 10.1016/B978-0-08-102203-0.00015-9.

[ref22] Kargozar S.; Montazerian M.; Hamzehlou S.; Kim H. W.; Baino F. Mesoporous Bioactive Glasses: Promising Platforms for Antibacterial Strategies. Acta Biomater. 2018, 81, 1–19. 10.1016/j.actbio.2018.09.052. 30273742

[ref23] Choi S. W.; Kim J. Recent Progress in Autocatalytic Ceria Nanoparticles-Based Translational Research on Brain Diseases. ACS Appl. Nano Mater. 2020, 3 (2), 1043–1062. 10.1021/acsanm.9b02243.

[ref24] Thakur N.; Manna P.; Das J. Synthesis and Biomedical Applications of Nanoceria, a Redox Active Nanoparticle. J. Nanobiotechnol. 2019, 17 (1), 84 10.1186/s12951-019-0516-9. PMC6617741 31291944

[ref25] Baino F.; Hamzehlou S.; Kargozar S. Bioactive Glasses: Where Are We and Where Are We Going?. J. Funct. Biomater. 2018, 9 (1), 25 10.3390/jfb9010025. PMC5872111 29562680

[ref26] Baino F.; Novajra G.; Miguez-Pacheco V.; Boccaccini A. R.; Vitale-Brovarone C. Bioactive Glasses: Special Applications Outside the Skeletal System. J. Non-Cryst. Solids 2016, 432, 15–30. 10.1016/j.jnoncrysol.2015.02.015.

[ref27] Reck R. Tissue Reactions to Glass Ceramics in the Middle Ear*. Clin. Otolaryngol. Allied Sci. 1981, 6 (1), 63–65. 10.1111/j.1365-2273.1981.tb01786.x. 7273453

[ref28] Ehrhardt G. J.; Day D. E. Therapeutic Use of 90Y Microspheres. Int. J. Radiat. Appl. Instrumentation. Part B. Nucl. Med. Biol. 1987, 14 (3), 233–242. 10.1016/0883-2897(87)90047-X. 3667306

[ref29] Gilchrist T.; Glasby M. A.; Healy D. M.; Kelly G.; Lenihan D. V.; McDowall K. L.; Miller I. A.; Myles L. M. In Vitro Nerve Repair - in Vivo. The Reconstruction of Peripheral Nerves by Entubulation with Biodegradeable Glass Tubes - a Preliminary Report. Br. J. Plast. Surg. 1998, 51 (3), 231–237. 10.1054/bjps.1997.0243. 9664883

[ref30] Gillette R. L.; Swaim S. F.; Sartin E. A.; Bradley D. M.; Coolman S. L. Effects of a Bioactive Glass on Healing of Closed Skin Wounds in Dogs. Am. J. Vet. Res. 2001, 62 (7), 1149–1153. 10.2460/ajvr.2001.62.1149. 11453494

[ref31] Verrier S.; Blaker J. J.; Maquet V.; Hench L. L.; Boccaccini A. R. PDLLA/Bioglass® Composites for Soft-Tissue and Hard-Tissue Engineering: An in Vitro Cell Biology Assessment. Biomaterials 2004, 25 (15), 3013–3021. 10.1016/j.biomaterials.2003.09.081. 14967534

[ref32] Bitar M.; C. Knowles J.; Lewis M. P.; Salih V. Soluble Phosphate Glass Fibres for Repair of Bone-Ligament Interface. J. Mater. Sci.: Mater. Med. 2005, 16 (12), 1131–1136. 10.1007/s10856-005-4718-3. 16362212

[ref33] Shah R.; Sinanan A. C. M.; Knowles J. C.; Hunt N. P.; Lewis M. P. Craniofacial Muscle Engineering Using a 3-Dimensional Phosphate Glass Fibre Construct. Biomaterials 2005, 26, 1497–1505. 10.1016/j.biomaterials.2004.04.049. 15522751

[ref34] Chen Q.; Jin L.; Cook W. D.; Mohn D.; Lagerqvist E. L.; Elliott D. A.; Haynes J. M.; Boyd N.; Stark W. J.; Pouton C. W.; Stanley E. G.; Elefanty A. G. Elastomeric Nanocomposites as Cell Delivery Vehicles and Cardiac Support Devices. Soft Matter 2010, 6 (19), 4715–4726. 10.1039/c0sm00213e.

[ref35] Kehoe S.; Abraham R.; Tonkopi E.; Boyd D. Abstract No. 74: Novel Radiopaque Embolic Agent for Uterine Fibroid Embolization: Determination of Radiopacity and Biological Evaluation; Cytocompatibility, Intracutaneous Reactivity and Local Effects after Implantation. J. Vasc. Interv. Radiol. 2012, 23 (3), S33 10.1016/j.jvir.2011.12.113.

[ref36] Joo N.-Y.; Knowles J. C.; Lee G.-S.; Kim J.-W.; Kim H.-W.; Son Y.-J.; Hyun J. K. Effects of Phosphate Glass Fiber-Collagen Scaffolds on Functional Recovery of Completely Transected Rat Spinal Cords. Acta Biomater. 2012, 8 (5), 1802–1812. 10.1016/j.actbio.2012.01.026. 22326790

[ref37] Chauhan N.; Mulcahy M. F.; Salem R.; Benson A. B. III; Boucher E.; Bukovcan J.; Cosgrove D.; Laframboise C.; Lewandowski R. J.; Master F.; El-Rayes B.; Strosberg J. R.; Sze D. Y.; Sharma R. A. TheraSphere Yttrium-90 Glass Microspheres Combined With Chemotherapy Versus Chemotherapy Alone in Second-Line Treatment of Patients With Metastatic Colorectal Carcinoma of the Liver: Protocol for the EPOCH Phase 3 Randomized Clinical Trial. JMIR Res. Protoc 2019, 8 (1), e11545 10.2196/11545. 30664496 PMC6354199

[ref38] Hench L. L. Bioceramics: From Concept to Clinic. J. Am. Ceram. Soc. 1991, 74 (7), 1487–1510. 10.1111/j.1151-2916.1991.tb07132.x.

[ref39] Yan X.; Yu C.; Zhou X.; Tang J.; Zhao D. Highly Ordered Mesoporous Bioactive Glasses with Superior in Vitro Bone-Forming Bioactivities. Angew. Chem., Int. Ed. 2004, 43 (44), 5980–5984. 10.1002/anie.200460598. 15547911

[ref40] López-Noriega A.; Arcos D.; Izquierdo-Barba I.; Sakamoto Y.; Terasaki O.; Vallet-Regí M. Ordered Mesoporous Bioactive Glasses for Bone Tissue Regeneration. Chem. Mater. 2006, 18 (13), 3137–3144. 10.1021/cm060488o.

[ref41] Du J.; Kokou L.; Rygel J. L.; Chen Y.; Pantano C. G.; Woodman R.; Belcher J. Structure of Cerium Phosphate Glasses: Molecular Dynamics Simulation. J. Am. Ceram. Soc. 2011, 94 (8), 2393–2401. 10.1111/j.1551-2916.2011.04514.x.

[ref42] Miniscalco W. J. Erbium-Doped Glasses for Fiber Amplifiers at 1500 Nm. J. Lightwave Technol. 1991, 9 (2), 234–250. 10.1109/50.65882.

[ref43] Iwanaga H. Emission Properties, Solubility, Thermodynamic Analysis and Nmr Studies of Rare-Earth Complexes with Two Different Phosphine Oxides. Materials 2010, 3 (8), 4080–4108. 10.3390/ma3084080. 28883322 PMC5445834

[ref44] Wei Y.; Zhao Z.; Jiao J.; Liu J.; Duan A.; Jiang G. Preparation of Ultrafine Ce-Based Oxide Nanoparticles and Their Catalytic Performances for Diesel Soot Combustion. J. Rare Earths 2014, 32 (2), 124–130. 10.1016/S1002-0721(14)60041-7.

[ref45] Tuler F. E.; Banús E. D.; Zanuttini M. A.; Miró E. E.; Milt V. G. Ceramic Papers as Flexible Structures for the Development of Novel Diesel Soot Combustion Catalysts. Chem. Eng. J. 2014, 246, 287–298. 10.1016/j.cej.2014.02.083.

[ref46] Nath P.; Tyagi B. S. Oxidation-Reduction Equilibrium in Glasses. Cent. Glas. Ceram. Res. Inst. Bull. 1972, 19 (4), 80–91.

[ref47] Pinet O.; Phalippou J.; Di Nardo C. Modeling the Redox Equilibrium of the Ce4+/Ce3+ Couple in Silicate Glass by Voltammetry. J. Non-Cryst. Solids 2006, 352 (50–51), 5382–5390. 10.1016/j.jnoncrysol.2006.08.034.

[ref48] Johnston W. D. Oxidation-Reduction Equilibria in Molten Na2O*SiO2. J. Am. Ceram. Soc. 1965, 48 (4), 184–190. 10.1111/j.1151-2916.1965.tb14709.x.

[ref49] Werner A. J. Colour Generation and Control in Glass, by C. R. Bamford. Elsevier Scientific Publishing Co., Amsterdam and New York, 1977. 224 Pp. Price, $34.95. Color Res. Appl. 1978, 3 (3), 156 10.1002/col.5080030317.

[ref50] Shimizu H.; Kitano T.; Nakayama K. Thermally Stimulated Depolarization Current Study on the Glass Transition of a Liquid Crystalline Copolyester. Japanese J. Appl. Physics, Part 2 Lett. 1996, 35 (Part 2, No. 2B), L231–L233. 10.1143/JJAP.35.L231.

[ref51] Paul A.; Mulholland M.; Zaman M. S. Ultraviolet Absorption of Cerium (Ill) and Cerium (IV) in Some s = Mple Glasses. J. Mater. Sci. 1976, 11, 2082–2086. 10.1007/BF02403358.

[ref52] Mohapatra G. K. D. A Spectroscopic Study of Ce3+ Ion in Calcium Metaphosphate Glass. Phys. Chem. Glasses 1998, 39 (1), 50–55.

[ref53] Lopez C.; Deschanels X.; Bart J. M.; Boubals J. M.; Den Auwer C.; Simoni E. Solubility of Actinide Surrogates in Nuclear Glasses. J. Nucl. Mater. 2003, 312 (1), 76–80. 10.1016/S0022-3115(02)01549-0.

[ref54] Leonelli C.; Lusvardi G.; Menabue L.; Tonelli M. Preliminary Experiments of in Situ Atomic Force Microscopy Observation of Hydroxyapatite Formation on Bioactive Glass Surface. J. Am. Ceram. Soc. 2002, 85 (2), 487 10.1111/j.1151-2916.2002.tb00118.x.

[ref55] Leonelli C.; Lusvardi G.; Malavasi G.; Menabue L.; Tonelli M. Synthesis and Characterization of Cerium-Doped Glasses and in Vitro Evaluation of Bioactivity. J. Non-Cryst. Solids 2003, 316 (2–3), 198 10.1016/S0022-3093(02)01628-9.

[ref56] Volf M. B. Chemical Approach to Glass; Elsevier, 1984.

[ref57] Park H. J.; Ryu B. K. Characterization and Catalytic Behavior of Cerium Oxide Doped into Aluminosilicophosphate Glasses. J. Ceram. Soc. Jpn. 2016, 124 (2), 155–159. 10.2109/jcersj2.15223.

[ref58] Zha J.; Roggendorf H. Sol-Gel Science, the Physics and Chemistry of Sol-Gel Processing, Ed. by C. J. Brinker and G. W. Scherer, Academic Press, Boston 1990, Xiv, 908 Pp., Bound-ISBN 0-12-134970-5. Adv. Mater. 1991, 3 (10), 522 10.1002/adma.19910031025.

[ref59] Li R.; Clark A. E.; Hench L. L. An Investigation of Bioactive Glass Powders by Sol-Gel Processing. J. Appl. Biomater. 1991, 2 (4), 231–239. 10.1002/jab.770020403. 10171144

[ref60] Mahony O.; Jones J. R. Porous Bioactive Nanostructured Scaffolds for Bone Regeneration: A Sol-Gel Solution. Nanomedicine 2008, 3 (2), 233–245. 10.2217/17435889.3.2.233. 18373428

[ref61] Peng T.-Y.; Tsai P.-Y.; Chen M.-S.; Mine Y.; Wu S.-H.; Chen C.-Y.; Lin D.-J.; Lin C.-K. Mesoporous Properties of Bioactive Glass Synthesized by Spray Pyrolysis with Various Polyethylene Glycol and Acid Additions. Polymers 2021, 13, 618 10.3390/polym13040618. 33670799 PMC7922486

[ref62] Oonishi H.; Kushitani S.; Yasukawa E.; Iwaki H.; Hench L. L.; Wilson J.; Tsuji E.; Sugihara T. Particulate Bioglass Compared with Hydroxyapatite as a Bone Graft Substitute. Clin. Orthop. Relat. Res. 1997, 334 (334), 316–325. 10.1097/00003086-199701000-00041. 9005929

[ref63] Sepulveda P.; Jones J. R.; Hench L. L. In Vitro Dissolution of Melt-Derived 45S5 and Sol-Gel Derived 58S Bioactive Glasses. J. Biomed. Mater. Res. 2002, 61 (2), 301–311. 10.1002/jbm.10207. 12007211

[ref64] Assefa Z.; Haire R. G.; Caulder D. L.; Shuh D. K. Correlation of the Oxidation State of Cerium in Sol-Gel Glasses as a Function of Thermal Treatment via Optical Spectroscopy and XANES Studies. Spectrochim. Acta, Part A 2004, 60 (8–9), 1873–1881. 10.1016/j.saa.2003.10.005. 15248963

[ref65] Beck J. S.; Vartuli J. C.; Roth W. J.; Leonowicz M. E.; Kresge C. T.; Schmitt K. D.; Chu C. T. W.; Olson D. H.; Sheppard E. W.; McCullen S. B.; Higgins J. B.; Schlenker J. L. A New Family of Mesoporous Molecular Sieves Prepared with Liquid Crystal Templates. J. Am. Chem. Soc. 1992, 114 (27), 10834–10843. 10.1021/ja00053a020.

[ref66] Kresge C. T.; Leonowicz M. E.; Roth W. J.; Vartuli J. C.; Beck J. S. Nature 1992, 359, 710–712. 10.1038/359710a0.

[ref67] Alothman Z. A. A Review: Fundamental Aspects of Silicate Mesoporous Materials. Materials 2012, 5 (12), 2874–2902. 10.3390/ma5122874.

[ref68] Brinker C. J. Evaporation-Induced Self-Assembly: Functional Nanostructures Made Easy. MRS Bull. 2004, 29 (9), 631–640. 10.1557/mrs2004.183.

[ref69] Migneco C.; Fiume E.; Verné E.; Baino F. A Guided Walk through the World of Mesoporous Bioactive Glasses (MBGs): Fundamentals, Processing, and Applications. Nanomaterials 2020, 10 (12), 2571 10.3390/nano10122571. PMC7767440 33371415

[ref70] Wu C.; Chang J. Mesoporous Bioactive Glasses: Structure Characteristics, Drug/Growth Factor Delivery and Bone Regeneration Application. Interface Focus 2012, 2 (3), 292–306. 10.1098/rsfs.2011.0121. 23741607 PMC3363021

[ref71] Vallet-Regí M. Ordered Mesoporous Materials in the Context of Drug Delivery Systems and Bone Tissue Engineering. Chem. - Eur. J. 2006, 12 (23), 5934–5943. 10.1002/chem.200600226. 16832799

[ref72] Salinas A. J.; Shruti S.; Malavasi G.; Menabue L.; Vallet-Regi M. Substitutions of Cerium, Gallium and Zinc in Ordered Mesoporous Bioactive Glasses. Acta Biomater. 2011, 7 (9), 3452–3458. 10.1016/j.actbio.2011.05.033. 21672640

[ref73] Chevalier J.; Gremillard L. Ceramics for Medical Applications: A Picture for the next 20 Years. J. Eur. Ceram. Soc. 2009, 29 (7), 1245–1255. 10.1016/j.jeurceramsoc.2008.08.025.

[ref74] Xia W.; Chang J. Well-Ordered Mesoporous Bioactive Glasses (MBG): A Promising Bioactive Drug Delivery System. J. Controlled Release 2006, 110 (3), 522–530. 10.1016/j.jconrel.2005.11.002. 16375986

[ref75] Wan Y.; Shi Y.; Zhao D. Supramolecular Aggregates as Templates: Ordered Mesoporous Polymers and Carbons. Chem. Mater. 2008, 20 (3), 932–945. 10.1021/cm7024125.

[ref76] Galarneau A.; Iapichella J.; Bonhomme K.; Di Renzo F.; Kooyman P.; Terasaki O.; Fajula F. Controlling the Morphology of Mesostructured Silicas by Pseudomorphic Transformation: A Route towards Applications. Adv. Funct. Mater. 2006, 16 (13), 1657–1667. 10.1002/adfm.200500825.

[ref77] Soler-Illia G. J. d. A. A.; Sanchez C.; Lebeau B.; Patarin J. Chemical Strategies to Design Textured Materials: From Microporous and Mesoporous Oxides to Nanonetworks and Hierarchical Structures. Chem. Rev. 2002, 102 (11), 4093–4138. 10.1021/cr0200062. 12428985

[ref78] García A.; Cicuéndez M.; Izquierdo-Barba I.; Arcos D.; Vallet-Regi M. Essential Role of Calcium Phosphate Heterogeneities in 2D-Hexagonal and 3D-Cubic SiO_2_-CaO-P_2_O_5_ Mesoporous Bioactive Glasses. Chem. Mater. 2009, 21 (22), 5474–5484. 10.1021/cm9022776.

[ref79] Li Z.; Chen D.; Tu B.; Zhao D. Synthesis and Phase Behaviors of Bicontinuous Cubic Mesoporous Silica from Triblock Copolymer Mixed Anionic Surfactant. Microporous Mesoporous Mater. 2007, 105 (1), 34–40. 10.1016/j.micromeso.2007.05.017.

[ref80] Nicolini V.; Malavasi G.; Lusvardi G.; Zambon A.; Benedetti F.; Cerrato G.; Valeri S.; Luches P. Mesoporous Bioactive Glasses Doped with Cerium: Investigation over Enzymatic-like Mimetic Activities and Bioactivity. Ceram. Int. 2019, 45 (16), 20910 10.1016/j.ceramint.2019.07.080.

[ref81] Atkinson I.; Anghel E. M.; Petrescu S.; Seciu A. M.; Stefan L. M.; Mocioiu O. C.; Predoana L.; Voicescu M.; Somacescu S.; Culita D.; Zaharescu M. Cerium-Containing Mesoporous Bioactive Glasses: Material Characterization, in Vitro Bioactivity, Biocompatibility and Cytotoxicity Evaluation. Microporous Mesoporous Mater. 2019, 276, 76–88. 10.1016/j.micromeso.2018.09.029.

[ref82] Kumar A.; Aditya A.; Murugavel S. Effect of Surfactant Concentration on Textural Characteristics and Biomineralization Behavior of Mesoporous Bioactive Glasses. Mater. Sci. Eng., C 2019, 96, 20–29. 10.1016/j.msec.2018.11.003. 30606526

[ref83] Zheng K.; Torre E.; Bari A.; Taccardi N.; Cassinelli C.; Morra M.; Fiorilli S.; Vitale-Brovarone C.; Iviglia G.; Boccaccini A. R. Antioxidant Mesoporous Ce-Doped Bioactive Glass Nanoparticles with Anti-Inflammatory and pro-Osteogenic Activities. Mater. Today Bio 2020, 5, 100041 10.1016/j.mtbio.2020.100041. PMC7083763 32211607

[ref84] Westhauser F.; Rehder F.; Decker S.; Kunisch E.; Moghaddam A.; Zheng K.; Boccaccini A. R. Ionic Dissolution Products of Cerium-Doped Bioactive Glass Nanoparticles Promote Cellular Osteogenic Differentiation and Extracellular Matrix Formation of Human Bone Marrow Derived Mesenchymal Stromal Cells. Biomed. Mater. 2021, 16, 035028 10.1088/1748-605X/abcf5f. 33260163

[ref85] Jones J. R.; Brauer D. S.; Hupa L.; Greenspan D. C. Bioglass and Bioactive Glasses and Their Impact on Healthcare. Int. J. Appl. Glas. Sci. 2016, 7 (4), 423–434. 10.1111/ijag.12252.

[ref86] Antoniac I. V. Handbook of Bioceramics and Biocomposites 2016, 1–1386. 10.1007/978-3-319-12460-5.

[ref87] Fiume E.; Barberi J.; Verné E.; Baino F. Bioactive Glasses: From Parent 45S5 Composition to Scaffold-Assisted Tissue-Healing Therapies. J. Funct. Biomater. 2018, 9 (1), 24 10.3390/jfb9010024. PMC5872110 29547544

[ref88] Hench L. L. The Story of Bioglass®. J. Mater. Sci.: Mater. Med. 2006, 17 (11), 967–978. 10.1007/s10856-006-0432-z. 17122907

[ref89] Kokubo T. Bioactive Glass Ceramics: Properties and Applications. Biomaterials 1991, 12 (2), 155–163. 10.1016/0142-9612(91)90194-F. 1878450

[ref90] Lusvardi G.; Malavasi G.; Menabue L.; Menziani M. C. Synthesis, Characterization, and Molecular Dynamics Simulation of Na2O-CaO-SiO2-ZnO Glasses. J. Phys. Chem. B 2002, 106 (38), 9753 10.1021/jp020321s.

[ref91] Nicolini V.; Malavasi G.; Menabue L.; Lusvardi G.; Benedetti F.; Valeri S.; Luches P. Cerium-Doped Bioactive 45S5 Glasses: Spectroscopic, Redox, Bioactivity and Biocatalytic Properties. J. Mater. Sci. 2017, 52 (15), 8845 10.1007/s10853-017-0867-2.

[ref92] Malavasi G.; Lusvardi G. Composition and Morphology Effects on Catalase Mimetic Activity of Potential Bioactive Glasses. Ceram. Int. 2020, 46 (16), 25854–25864. 10.1016/j.ceramint.2020.07.067.

[ref93] Ershad M.; Ali A.; Mehta N. S.; Singh R. K.; Singh S. K.; Pyare R. Mechanical and Biological Response of (CeO2+La2O3)-Substituted 45S5 Bioactive Glasses for Biomedical Application. J. Aust. Ceram. Soc. 2020, 56 (4), 1243–1252. 10.1007/s41779-020-00471-3.

[ref94] Nicolini V.; Varini E.; Malavasi G.; Menabue L.; Menziani M. C.; Lusvardi G.; Pedone A.; Benedetti F.; Luches P. The Effect of Composition on Structural, Thermal, Redox and Bioactive Properties of Ce-Containing Glasses. Mater. Des. 2016, 97, 73 10.1016/j.matdes.2016.02.056.

[ref95] Nicolini V.; Caselli M.; Ferrari E.; Menabue L.; Lusvardi G.; Saladini M.; Malavasi G. SiO_2_-CaO-P_2_O_5_ Bioactive Glasses: A Promising Curcuminoids Delivery System. Materials 2016, 9 (4), 290 10.3390/ma9040290. PMC5502983 28773414

[ref96] Lusvardi G.; Stabellini F. S.; Salvatori R. P_2_O_5_-Free Cerium Containing Glasses: Bioactivity and Cytocompatibility Evaluation. Materials 2019, 12 (19), 3267 10.3390/ma12193267. PMC6803907 31597232

[ref97] Shruti S.; Salinas A. J.; Lusvardi G.; Malavasi G.; Menabue L.; Vallet-Regi M. Mesoporous Bioactive Scaffolds Prepared with Cerium-, Gallium- and Zinc-Containing Glasses. Acta Biomater. 2013, 9 (1), 4836 10.1016/j.actbio.2012.09.024. 23026489

[ref98] Shruti S.; Salinas A. J.; Malavasi G.; Lusvardi G.; Menabue L.; Ferrara C.; Mustarelli P.; Vallet-Regì M. Structural and in Vitro Study of Cerium, Gallium and Zinc Containing Sol-Gel Bioactive Glasses. J. Mater. Chem. 2012, 22 (27), 13698 10.1039/c2jm31767b.

[ref99] Shruti S.; Salinas A. J.; Ferrari E.; Malavasi G.; Lusvardi G.; Doadrio A. L.; Menabue L.; Vallet-Regi M. Curcumin Release from Cerium, Gallium and Zinc Containing Mesoporous Bioactive Glasses. Microporous Mesoporous Mater. 2013, 180, 92 10.1016/j.micromeso.2013.06.014.

[ref100] Goh Y.-F.; Alshemary A. Z.; Akram M.; Abdul Kadir M. R.; Hussain R. In-Vitro Characterization of Antibacterial Bioactive Glass Containing Ceria. Ceram. Int. 2014, 40 (1PA), 729–737. 10.1016/j.ceramint.2013.06.062.

[ref101] Shruti S.; Andreatta F.; Furlani E.; Marin E.; Maschio S.; Fedrizzi L. Cerium, Gallium and Zinc Containing Mesoporous Bioactive Glass Coating Deposited on Titanium Alloy. Appl. Surf. Sci. 2016, 378, 216–223. 10.1016/j.apsusc.2016.03.209.

[ref102] Zhang Y.; Jiang F.; Luan J.; Zhou X.; Wu Z.; Li M.; Hong Z. Surface Properties of Ce-TZP/Al_2_O_3_ Composite Ceramics by Coating Mesoporous Bioactive Glass. Composites, Part B 2019, 164, 499–507. 10.1016/j.compositesb.2019.01.068.

[ref103] Deliormanli A. M. Electrospun Cerium and Gallium-Containing Silicate Based 13–93 Bioactive Glass Fibers for Biomedical Applications. Ceram. Int. 2016, 42, 897–906. 10.1016/j.ceramint.2015.09.016.

[ref104] Deliormanli A. M.; Seda Vatansever H.; Yesil H.; Ozdal-Kurt F. In Vivo Evaluation of Cerium, Gallium and Vanadium-Doped Borate-Based Bioactive Glass Scaffolds Using Rat Subcutaneous Implantation Model. Ceram. Int. 2016, 42 (10), 11574–11583. 10.1016/j.ceramint.2016.04.033.

[ref105] Deliormanir A. M.; Yildinm M. Sol-Gel Synthesis of 13–93 Bioactive Glass Powders Containing Therapeutic Agents. J. Aust. Ceram. Soc. 2016, 52 (2), 9–19.

[ref106] Massera J.; Vassallo-Breillot M.; Torngren B.; Glorieux B.; Hupa L. Effect of CeO_2_ Doping on Thermal, Optical, Structural and in Vitro Properties of a Phosphate Based Bioactive Glass. J. Non-Cryst. Solids 2014, 402, 28–35. 10.1016/j.jnoncrysol.2014.05.006.

[ref107] Goh Y.-f.; Akram M.; Alshemary A.; Hussain R. Antibacterial Polylactic Acid/Chitosan Nanofibers Decorated with Bioactive Glass. Appl. Surf. Sci. 2016, 387, 1–7. 10.1016/j.apsusc.2016.06.054.

[ref108] Placek L. M.; Keenan T. J.; Wren A. W. Bioactivity of Y_2_O_3_ and CeO_2_ Doped SiO_2_-SrO-Na_2_O Glass-Ceramics. J. Biomater. Appl. 2016, 31 (2), 165–180. 10.1177/0885328216651392. 27231265

[ref109] Sobhanachalam P.; Ravi Kumar V.; Venkatramaiah N.; Gandhi Y.; Veeraiah N. Synthesis and in Vitro Characterization of Cerium Oxide Mixed Calcium Oxy Fluoro Borophosphate Bioactive Glasses by Means of Spectroscopic Studies. J. Non-Cryst. Solids 2018, 498, 422–429. 10.1016/j.jnoncrysol.2018.02.035.

[ref110] Youness R. A.; Taha M. A.; El-Kheshen A. A.; El-Faramawy N.; Ibrahim M. In Vitro Bioactivity Evaluation, Antimicrobial Behavior and Mechanical Properties of Cerium-Containing Phosphate Glasses. Mater. Res. Express 2019, 6 (7), 075212 10.1088/2053-1591/ab15b5.

[ref111] Kaur P.; Singh K. J.; Yadav A. K.; Kaur S.; Kaur R.; Kaur S. Growth of Bone like Hydroxyapatite and Cell Viability Studies on CeO_2_ Doped CaO-P_2_O_5_-MgO-SiO_2_ Bioceramics. Mater. Chem. Phys. 2020, 243, 122352 10.1016/j.matchemphys.2019.122352.

[ref112] Goller G.; Akin I. Effect of CeO2 Addition on In-Vitro Bioactivity Properties of K-Mica-Fluorapatite Based Glass Ceramics. Key Eng. Mater. 2007, 361–363, 261–264. 10.4028/www.scientific.net/KEM.361-363.261.

[ref113] Deliormanlı A. M. Synthesis and Characterization of Cerium- and Gallium-Containing Borate Bioactive Glass Scaffolds for Bone Tissue Engineering. J. Mater. Sci.: Mater. Med. 2015, 26 (2), 67 10.1007/s10856-014-5368-0. 25631259

[ref114] Sherief M. A.; Hanna A. A.; El-kheshen A. A.; Abd El Aty A. A. Studies on the Bioactive Effects of Incorporate Some Rare Earth Elements into Basic Glass Materials. Rasayan J. Chem. 2016, 9 (3), 531–543.

[ref115] Farag M. M.; Al-Rashidy Z. M.; Ahmed M. M. In Vitro Drug Release Behavior of Ce-Doped Nano-Bioactive Glass Carriers under Oxidative Stress. J. Mater. Sci.: Mater. Med. 2019, 30 (2), 1–15. 10.1007/s10856-019-6220-3. 30671708

[ref116] Varini E.; Sanchez-Salcedo S.; Malavasi G.; Lusvardi G.; Vallet-Regí M.; Salinas A. J. Cerium (III) and (IV) Containing Mesoporous Glasses/Alginate Beads for Bone Regeneration: Bioactivity, Biocompatibility and Reactive Oxygen Species Activity. Mater. Sci. Eng., C 2019, 105, 109971 10.1016/j.msec.2019.109971. PMC6736678 31507308

[ref117] Yufeng Z.; Zhenghua W.; Xiaorong W.; Lemei D.; Yijun C. Mobility of the Rare Earth Elements with Acid Rainwater Leaching in the Soil Column. Bull. Environ. Contam. Toxicol. 2001, 67 (3), 399–407. 10.1007/s001280138. 11479670

[ref118] Zhenghua W.; Xiaorong W.; Yufeng Z.; Lemei D.; Yijun C. Effects of Apatite and Calcium Oxyphosphate on Speciation and Bioavailability of Exogenous Rare Earth Elements in the Soil-Plant System. Chem. Speciation Bioavailability 2001, 13 (2), 49–56. 10.3184/095422901783726816.

[ref119] Clark A. E.; Pantano C. G.; Hench L. L. Auger Spectroscopic Analysis of Bioglass Corrosion Films. J. Am. Ceram. Soc. 1976, 59 (1–2), 37–39. 10.1111/j.1151-2916.1976.tb09382.x.

[ref120] Sanders D. M.; Hench L. L. Mechanisms of Glass Corrosion. J. Am. Ceram. Soc. 1973, 56 (7), 373–377. 10.1111/j.1151-2916.1973.tb12689.x.

[ref121] Maçon A. L. B.; Kim T. B.; Valliant E. M.; Goetschius K.; Brow R. K.; Day D. E.; Hoppe A.; Boccaccini A. R.; Kim I. Y.; Ohtsuki C.; Kokubo T.; Osaka A.; Vallet-Regí M.; Arcos D.; Fraile L.; Salinas A. J.; Teixeira A. V.; Vueva Y.; Almeida R. M.; Miola M.; Vitale-Brovarone C.; Verné E.; Höland W.; Jones J. R. A Unified in Vitro Evaluation for Apatite-Forming Ability of Bioactive Glasses and Their Variants. J. Mater. Sci.: Mater. Med. 2015, 26 (2), 1–10. 10.1007/s10856-015-5403-9. 25665841

[ref122] Goonoo N.; Bhaw-Luximon A.; Jhurry D. In Vitro and in Vivo Cytocompatibility of Electrospun Nanofiber Scaffolds for Tissue Engineering Applications. RSC Adv. 2014, 4 (60), 31618–31642. 10.1039/C4RA05218H.

[ref123] Deliormanlı A. M. Electrospun Cerium and Gallium-Containing Silicate Based 13–93 Bioactive Glass Fibers for Biomedical Applications. Ceram. Int. 2016, 42 (1), 897–906. 10.1016/j.ceramint.2015.09.016.

[ref124] Jones C. F.; Grainger D. W. In Vitro Assessments of Nanomaterial Toxicity. Adv. Drug Delivery Rev. 2009, 61 (6), 438–456. 10.1016/j.addr.2009.03.005. PMC2763955 19383522

[ref125] Prefac G.-A.; Milea M.-L.; Vadureanu A.-M.; Muraru S.; Dobrin D.-I.; Isopencu G.-O.; Jinga S.-I.; Raileanu M.; Bacalum M.; Busuioc C. CeO_2_ Containing Thin Films as Bioactive Coatings for Orthopaedic Implants. Coatings 2020, 10, 642 10.3390/coatings10070642.

[ref126] Saatchi A.; Arani A. R.; Moghanian A.; Mozafari M. Synthesis and Characterization of Electrospun Cerium-Doped Bioactive Glass/Chitosan/Polyethylene Oxide Composite Scaffolds for Tissue Engineering Applications. Ceram. Int. 2021, 47 (1), 260–271. 10.1016/j.ceramint.2020.08.130.

[ref127] Anesi A.; Malavasi G.; Chiarini L.; Salvatori R.; Lusvardi G. Cell Proliferation to Evaluate Preliminarily the Presence of Enduring Self-Regenerative Antioxidant Activity in Cerium Doped Bioactive Glasses. Materials 2020, 13 (10), 2297 10.3390/ma13102297. PMC7288167 32429291

[ref128] Malavasi G.; Salvatori R.; Zambon A.; Lusvardi G.; Rigamonti L.; Chiarini L.; Anesi A. Cytocompatibility of Potential Bioactive Cerium-Doped Glasses Based on 45S5. Materials 2019, 12 (4), 594 10.3390/ma12040594. PMC6416737 30781522

[ref129] Morais D. S.; Fernandes S.; Gomes P. S.; Fernandes M. H.; Sampaio P.; Ferraz M. P.; Santos J. D.; Lopes M. A.; Sooraj Hussain N. Novel Cerium Doped Glass-Reinforced Hydroxyapatite with Antibacterial and Osteoconductive Properties for Bone Tissue Regeneration. Biomed. Mater. 2015, 10 (5), 055008 10.1088/1748-6041/10/5/055008. 26391473

[ref130] Bellucci D.; Salvatori R.; Anesi A.; Chiarini L.; Cannillo V. SBF Assays, Direct and Indirect Cell Culture Tests to Evaluate the Biological Performance of Bioglasses and Bioglass-Based Composites: Three Paradigmatic Cases. Mater. Sci. Eng., C 2019, 96, 757–764. 10.1016/j.msec.2018.12.006. 30606588

[ref131] Akin I.; Goller G. Effect of CeO_2_ Addition on Crystallization Behavior, Bioactivity and Biocompatibility of Potassium Mica and Fluorapatite Based Glass Ceramics. J. Ceram. Soc. Jpn. 2009, 117 (1367), 787–792. 10.2109/jcersj2.117.787.

[ref133] Baino F.; Ferraris S.; Miola M.; Verné E.; Evans I.; Bretcanu O. Multifunctional Bioactive Glasses and Glass-Ceramics: Beyond ‘Traditional’ Bioactivity; Elsevier Ltd., 2018; 10.1016/B978-0-08-102196-5.00002-1.

[ref134] Hu S.; Chang J.; Liu M.; Ning C. Study on Antibacterial Effect of 45S5 Bioglass®. J. Mater. Sci.: Mater. Med. 2009, 20 (1), 281–286. 10.1007/s10856-008-3564-5. 18763024

[ref135] Zeyons O.; Thill A.; Chauvat F.; Menguy N.; Cassier-Chauvat C.; Oréar C.; Daraspe J.; Auffan M.; Rose J.; Spalla O. Direct and Indirect CeO_2_ Nanoparticles Toxicity for Escherichia Coli and Synechocystis. Nanotoxicology 2009, 3 (4), 284–295. 10.3109/17435390903305260.

[ref136] Begum S.; Johnson W. E.; Worthington T.; Martin R. A. The Influence of pH and Fluid Dynamics on the Antibacterial Efficacy of 45S5 Bioglass. Biomed. Mater. 2016, 11 (1), 015006 10.1088/1748-6041/11/1/015006. 26836582

[ref137] Drago L.; Toscano M.; Bottagisio M. Recent Evidence on Bioactive Glass Antimicrobial and Antibiofilm Activity: A Mini-Review. Materials 2018, 11 (2), 326 10.3390/ma11020326. PMC5849023 29495292

[ref138] Sobek J. M.; Talburt D. E. Effects of the Rare Earth Cerium on Escherichia Coli. J. Bacteriol. 1968, 95 (1), 47–51. 10.1128/jb.95.1.47-51.1968. 4866102 PMC251970

[ref139] Qi M.; Li W.; Zheng X.; Li X.; Sun Y.; Wang Y.; Li C.; Wang L. Cerium and Its Oxidant-Based Nanomaterials for Antibacterial Applications: A State-of-the-Art Review. Front. Mater. 2020, 7 (July), 1–26. 10.3389/fmats.2020.00213.

[ref140] Thill A.; Zeyons O.; Spalla O.; Chauvat F.; Rose J.; Auffan M.; Flank A. M. Cytotoxicity of CeO2 Nanoparticles for Escherichia Coli. Physico-Chemical Insight of the Cytotoxicity Mechanism. Environ. Sci. Technol. 2006, 40 (19), 6151–6156. 10.1021/es060999b. 17051814

[ref141] Zhang M.; Zhang C.; Zhai X.; Luo F.; Du Y.; Yan C. Antibacterial Mechanism and Activity of Cerium Oxide Nanoparticles. Sci. China Mater. 2019, 62 (11), 1727–1739. 10.1007/s40843-019-9471-7.

[ref142] Kargozar S.; Montazerian M.; Hamzehlou S.; Kim H.-W.; Baino F. Mesoporous Bioactive Glasses (MBGs): Promising Platforms for Antibacterial Strategies. Acta Biomater. 2018, 81, 1–19. 10.1016/j.actbio.2018.09.052. 30273742

[ref143] Rahimi R.; Nikfar S.; Larijani B.; Abdollahi M. A Review on the Role of Antioxidants in the Management of Diabetes and Its Complications. Biomed. Pharmacother. 2005, 59 (7), 365–373. 10.1016/j.biopha.2005.07.002. 16081237

[ref144] Rosenfeldt F.; Wilson M.; Lee G.; Kure C.; Ou R.; Braun L.; de Haan J. Oxidative Stress in Surgery in an Ageing Population: Pathophysiology and Therapy. Exp. Gerontol. 2013, 48, 45 10.1016/j.exger.2012.03.010. 22465624

[ref145] Kelly F. J. Oxidative stress: its role in air pollution and adverse health effects. Occup. Environ. Med. 2003, 60, 612–616. 10.1136/oem.60.8.612. 12883027 PMC1740593

[ref146] Karakoti A.; Singh S.; Dowding J. M.; Seal S.; Self W. T. Redox-Active Radical Scavenging Nanomaterials. Chem. Soc. Rev. 2010, 39 (11), 4422–4432. 10.1039/b919677n. 20717560

[ref147] Korsvik C.; Patil S.; Seal S.; Self W. T. Superoxide Dismutase Mimetic Properties Exhibited by Vacancy Engineered Ceria Nanoparticles. Chem. Commun. 2007, (10), 1056–1058. 10.1039/b615134e. 17325804

[ref148] Bergandi L.; Aina V.; Garetto S.; Malavasi G.; Aldieri E.; Laurenti E.; Matera L.; Morterra C.; Ghigo D. Fluoride-Containing Bioactive Glasses Inhibit Pentose Phosphate Oxidative Pathway and Glucose 6-Phosphate Dehydrogenase Activity in Human Osteoblasts. Chem.-Biol. Interact. 2010, 183 (3), 405–415. 10.1016/j.cbi.2009.11.021. 19945446

[ref149] Milkovic L.; Siems W.; Siems R.; Zarkovic N. Oxidative Stress and Antioxidants in Carcinogenesis and Integrative Therapy of Cancer. Curr. Pharm. Des. 2014, 20, 6529–6542. 10.2174/1381612820666140826152822. 25341930

[ref150] Nicolini V.; Gambuzzi E.; Malavasi G.; Menabue L.; Menziani M. C.; Lusvardi G.; Pedone A.; Benedetti F.; Luches P.; D’Addato S.; Valeri S. Evidence of Catalase Mimetic Activity in Ce^3+^/Ce^4+^ Doped Bioactive Glasses. J. Phys. Chem. B 2015, 119 (10), 4009 10.1021/jp511737b. 25710332

[ref151] Benedetti F.; Luches P.; D’Addato S.; Valeri S.; Nicolini V.; Pedone A.; Menziani M. C.; Malavasi G. Structure of Active Cerium Sites within Bioactive Glasses. J. Am. Ceram. Soc. 2017, 100 (11), 5086–5095. 10.1111/jace.15049.

[ref152] Benedetti F.; Amidani L.; Pelli Cresi J. S.; Boscherini F.; Valeri S.; D’Addato S.; Nicolini V.; Malavasi G.; Luches P. Role of Cerium Oxide in Bioactive Glasses during Catalytic Dissociation of Hydrogen Peroxide. Phys. Chem. Chem. Phys. 2018, 20 (36), 23507–23514. 10.1039/C8CP02271B. 30183019

[ref153] Ukeda H.; Kawana D.; Maeda S.; Sawamura M. Spectrophotometric Assay for Superoxide Dismutase Based on the Reduction of Highly Water-Soluble Tetrazolium Salts by Xanthine-Xanthine Oxidase. Biosci., Biotechnol., Biochem. 1999, 63 (3), 485–488. 10.1271/bbb.63.485. 27393255

[ref154] Kargozar S.; Lotfibakhshaiesh N.; Ai J.; Samadikuchaksaraie A.; Hill R. G.; Shah P. A.; Milan P. B.; Mozafari M.; Fathi M.; Joghataei M. T. Synthesis, Physico-Chemical and Biological Characterization of Strontium and Cobalt Substituted Bioactive Glasses for Bone Tissue Engineering. J. Non-Cryst. Solids 2016, 449, 133 10.1016/j.jnoncrysol.2016.07.025.

[ref155] Kargozar S.; Baino F.; Hamzehlou S.; Hill R. G.; Mozafari M. Bioactive Glasses: Sprouting Angiogenesis in Tissue Engineering. Trends Biotechnol. 2018, 36, 430–444. 10.1016/j.tibtech.2017.12.003. 29397989

[ref156] Zhang Q.; Ge K.; Ren H.; Zhang C.; Zhang J. Effects of Cerium Oxide Nanoparticles on the Proliferation, Osteogenic Differentiation and Adipogenic Differentiation of Primary Mouse Bone Marrow Stromal Cells *In Vitro*. J. Nanosci. Nanotechnol. 2015, 15 (9), 6444 10.1166/jnn.2015.10709. 26716198

[ref157] Kurtuldu F.; Mutlu N.; Michálek M.; Zheng K.; Masar M.; Liverani L.; Chen S.; Galusek D.; Boccaccini A. R. Cerium and Gallium Containing Mesoporous Bioactive Glass Nanoparticles for Bone Regeneration: Bioactivity, Biocompatibility and Antibacterial Activity. Mater. Sci. Eng., C 2021, 124, 112050 10.1016/j.msec.2021.112050. 33947544

[ref158] Xiang J.; Li J.; He J.; Tang X.; Dou C.; Cao Z.; Yu B.; Zhao C.; Kang F.; Yang L.; Dong S.; Yang X. Cerium Oxide Nanoparticle Modified Scaffold Interface Enhances Vascularization of Bone Grafts by Activating Calcium Channel of Mesenchymal Stem Cells. ACS Appl. Mater. Interfaces 2016, 8 (7), 4489 10.1021/acsami.6b00158. 26824825

[ref159] Lu B.; Zhu D.-Y.; Yin J.-H.; Xu H.; Zhang C.-Q.; Ke Q.-F.; Gao Y.-S.; Guo Y.-P. Incorporation of Cerium Oxide in Hollow Mesoporous Bioglass Scaffolds for Enhanced Bone Regeneration by Activating the ERK Signaling Pathway. Biofabrication 2019, 11 (2), 025012 10.1088/1758-5090/ab0676. 30754024

